# Dystrophin Restorative and Compensatory Gene Addition Therapies for Duchenne Muscular Dystrophy: Could CRISPRa Provide a Realistic Alternative?

**DOI:** 10.3390/muscles4040052

**Published:** 2025-11-10

**Authors:** Zakaria Rostamitehrani, Rida Javed, Linda Popplewell

**Affiliations:** 1 National Horizons Centre, School of Health and Life Sciences, Teesside University, Darlington DL1 1HG, UK; l.popplewell@tees.ac.uk; 2 Department of Biological Sciences, School of Life Sciences and the Environment, Royal Holloway University of London, Egham TW20 0EX, UK; rida.javed.2021@live.rhul.ac.uk

**Keywords:** Duchenne muscular dystrophy, CRISPRa, utrophin, follistatin, Klotho, GALGT2, AAV, micro-dystrophin

## Abstract

Duchenne muscular dystrophy (DMD), which results from mutations that disrupt the expression of dystrophin proteins, is characterized by progressive muscle fiber wasting and the development of skeletal muscle fibrosis. The severe pathology leads to loss of ambulation, respiratory insufficiency, cardiomyopathy, and early death in patients. Dystrophin-focused therapies based on adeno-associated viral (AAV) vector-mediated gene addition, antisense oligonucleotide-induced repair of the transcript reading frame, and chemically driven stop codon readthrough have been conditionally approved for use in subsets of patients. From trials, it is apparent that these therapies act to stabilize the disease phenotype rather than improve it significantly, meaning that early treatment results in better outcomes. AAV-mediated delivery of a form of utrophin, a structural and functional homolog of dystrophin, GALGT2, a sarcolemmal stabilizer, and Klotho, the anti-aging hormone that is silenced in a mouse model of DMD as a result of the disease pathology, have been explored in preclinical compensatory gene addition studies. Recombinant follistatin protein has been used to target the fibrosis seen. An all-in-one type of therapy is likely to provide a synergistic effect such that efficacy of the dystrophin restoration strategy would be improved. For this, CRISPRa could hold potential through the targeting of multiple relevant genes simultaneously. The suitability of targeting these genes will be discussed, as will the stages of the development of CRISPRa for DMD. A perspective on the future prospects of CRISPRa in relation to likely issues that would need addressing and how they may be overcame will be given.

## 1. Introduction

Muscular dystrophies (MDs) are characterized by progressive muscle fiber wasting and muscle fibrosis development [1]. Ultimately, muscular atrophy causes suffering in patients with MD and shortens their lifespan. There are many different types of MD, but its overall diagnostic occurrence is 19.8–25.1 per 100,000 people per year [2]. The most common types of MD are Myotonic dystrophy (0.5–18.1 per 100,000), Duchenne muscular dystrophy (DMD) (1.7–4.2 per 100,000), and facioscapulohumeral muscular dystrophy (3.2–12.5 per 100,000) [3,4,5,6,7]. DMD is the most severe type of MD and is caused by out-of-frame mutations on the X-linked *DMD* gene and the loss of functional dystrophin protein expression [8,9].

## 2. Pathology of DMD

**Figure 1:**
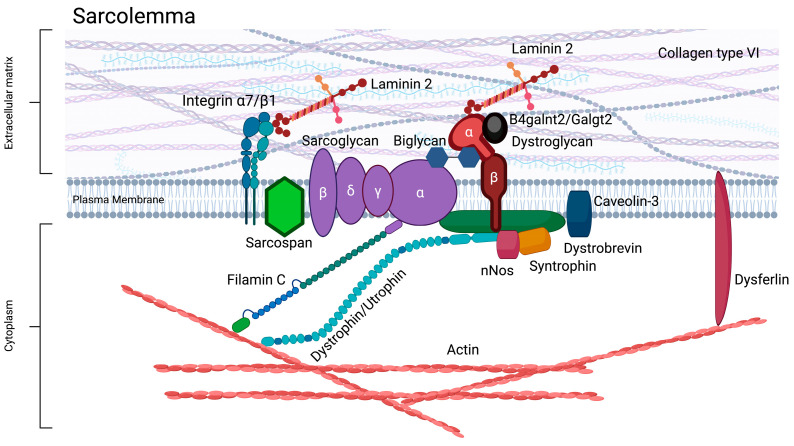
Sarcolemma and dystrophin complex. This figure illustrates the interaction between dystrophin, actin, and other dystrophin complex proteins. Utrophin, the fetal homolog of dystrophin, is also shown [15,16]. “Created in BioRender. Rt, Z and Javed, R. (2025) https://BioRender.com/ofjbw2n (accessed on 11 October 2025).

The dystrophin protein normally forms a complex linkage between actin filaments within the sarcomere and the sarcolemma, interacting specifically with proteins such as Dystroglycan and Dystrobrevin, as depicted in Figure 1 [8]. This multi-protein complex, known as the dystrophin-associated glycoprotein complex (DGC), serves critical functions in stabilizing the sarcolemma and transmitting the force generated during muscle contractions, protecting the sarcolemma from damage caused by mechanical stress, thereby preserving the structural integrity and functional capacity of muscle fibers [10]. When dystrophin is not present or is dysfunctional, the muscle cell membrane ruptures during contraction and allows an overloading influx of Ca^2+^ ions into the fiber’s cytoplasm that activates apoptotic and necrotic pathways [11]. The constant muscle cell death exhausts the regenerative capacity of muscle stem cells, leading to the progressive replacement of muscle fibers by adipose and fibrotic tissue [12,13,14]. DMD patients typically manifest early symptoms of skeletal muscle degeneration by the age of five, and typically have a life expectancy of less than 35 years due to severe and progressive muscle deterioration, respiratory insufficiency, and cardiomyopathy.

## 3. Conditionally Approved Gene and Gene-Targeting Therapies for DMD

Currently, there are no cures available for DMD, but there are several treatment options that can help manage the symptoms and slow the progression of the disease [17]. These treatments include corticosteroid medications to reduce muscle inflammation, muscle relaxant such as Vecuronium to manage muscle spasms, physical therapy to maintain muscle strength and function, and assistive devices to help with mobility [17,18,19,20]. In some cases, surgery may be necessary to correct deformities or improve movement [21]. In recent years, several dystrophin-based gene therapies have promised a better and longer quality of life for these patients.

### 3.1. Stop Codon Readthrough

Nearly 15% of DMD cases are due to nonsense mutations which result in the premature termination of dystrophin mRNA translation so no dystrophin protein is produced [22]. Chemicals that allow readthrough of these premature termination codons and the expression of a functional dystrophin protein have been developed [23]. An example of this readthrough approach is Ataluren (Translarna) which delays the progression of DMD in patients and is a European Medicines Authority (EMA) conditionally approved medicine [22,23]. Stop codon readthrough is mutation-specific therapy and it requires daily oral drug administration [22]. In trial, Ataluren led to a mean change of 11% in expression of dystrophin after the treatment of DMD patients [24]. In animal studies, it has been established that for therapeutic efficacy to be realized, the restoration of dystrophin to 20% of WT levels is required [25]. The effects of the drug on respiratory and cardiac parameters have not led to satisfactory results [26]. Two recent studies by the EMA Human Medicine Committee (CHMP) compared the clinical status of patients treated with Translarna to patient registries and patients treated with a placebo. The conclusion of these studies was that the effectiveness of Translarna could not be confirmed. As a result, the CHMP has recommended that the EMA does not renew its market approval of Translarna [27].

### 3.2. Exon Skipping

**Figure 2:**
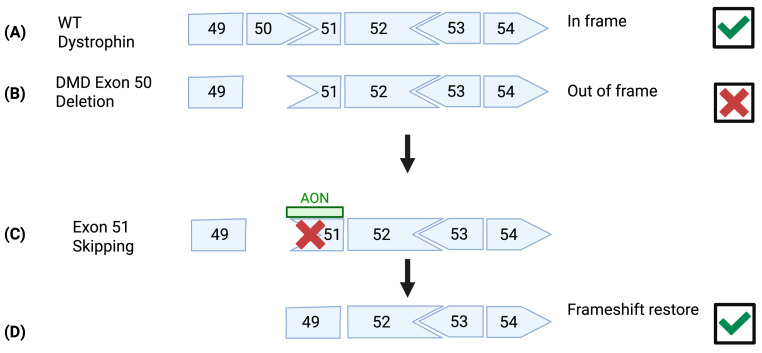
Exon skipping mechanism. (**A**) In frame dystrophin pre-mRNA. (**B**) Deletion of exon 50 putting dystrophin pre-mRNA out of frame. (**C**) Targeted *DMD* exon 51 skipping via AOs. (**D**) Frameshift of the dystrophin pre-mRNA is restored which leads to the production of truncated dystrophin. “Created in BioRender. Rt, Z. (2025) https://BioRender.com/uvi0q97 (accessed on 11 October 2025)”.

Certain antisense oligonucleotides (AOs) that induce the skipping of out-of-frame exons that neighbor particular mutations during the maturation of pre-mRNA are US Food and Drug Administration (FDA) conditionally approved treatments for DMD. This exon skipping restores the transcript reading frame and allows the expression of an internally truncated protein, as shown in Figure 2 [28]. This can help improve muscle function and slow the progression of the disease [17].

AOs for exon skipping require weekly intravenous administration and it is a mutation-based therapy [29]. The mutation hotspots for DMD are within exons 2–20 and 45–55 of the *DMD* gene, with 65% of patients carrying mutations within the latter region, making these highly applicable exons to target AOs [30,31]. Despite very low levels of dystrophin protein restoration, Eteplirsen for exon 51 skipping, Golodirsen, and Viltolarsen, which both target exon 53 and Casimersen for exon 45 skipping, have all been conditionally approved by FDA for use in patients carrying appropriate mutations [30]. AO therapies slow fibrosis and disease progression [32]. However, they do not reverse the effects of the disease in terms of muscle pathology [33]. Therefore, exon skipping has been shown to have the best therapeutic effect in younger patients [34]. Eteplirsen, the first approved AO for genetic disease, has been shown to improve long-term ambulatory function and to delay pulmonary decline [35,36]. Clinical trials are now underway to assess improvements in delivery and efficacy using various targeting moieties.

### 3.3. Microdystrophin Gene Addition

**Figure 3:**
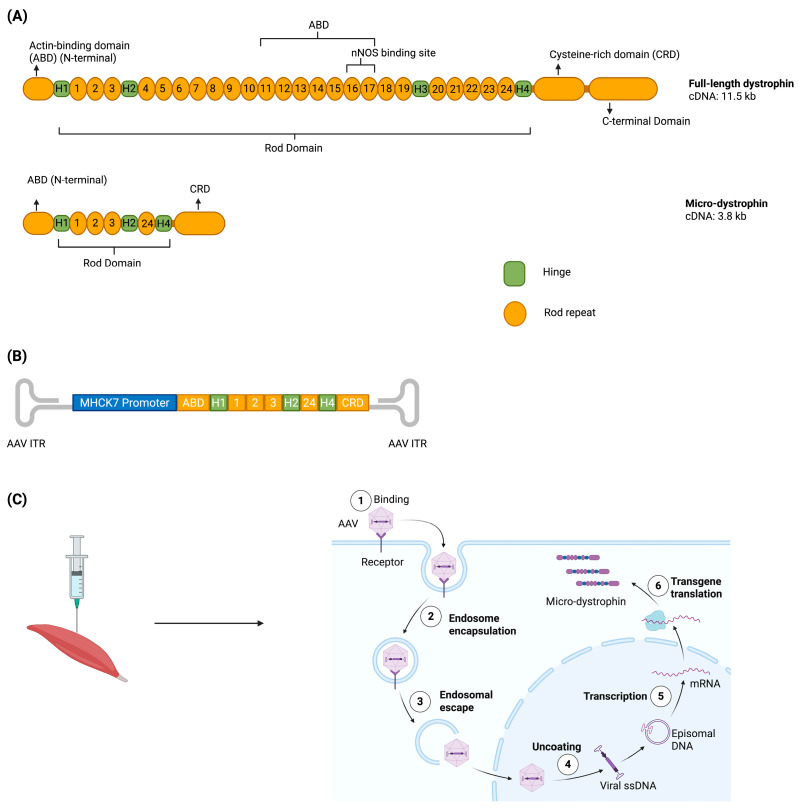
Micro-dystrophin therapy. (**A**) shows cDNAs and genomic structural maps of full-length dystrophin and micro-dystrophin [39]. (**B**) shows the linear AAV-micro-dystrophin plasmid map [56]. (**C**) shows the AAV vector’s muscular injection into a patient’s muscles which leads to the production of micro-dystrophin with the patient’s muscle cells. “Created with BioRender.com with part C adapted from “AAV Vector Infection”, by BioRender.com (2024), retrieved from https://app.biorender.com/biorender-templates (accessed on 15 June 2024). Created in BioRender. Rt, Z. (2025) https://BioRender.com/my8oybt (accessed on 11 October 2025)”.

Microdystrophin gene transfer using recombinant adeno-associated viral (rAAV) vectors has been recently conditionally approved by the FDA [37,38]. Since rAAV vectors have a limited packaging capacity, micro-dystrophin cDNAs encoding internally truncated, but semi-functional, dystrophin proteins have been developed (Figure 3) [37,39]. Similar to exon skipping, micro-dystrophin is shown in trials to stabilize DMD progression by reducing or slowing down muscle wasting and fibrosis, while increasing muscle mass and strength in patients [40,41,42].

Studies in *mdx* and golden retriever muscular dystrophy (GRMD) dog models have shown the efficiency of different microdystrophin constructs in treating DMD. In these studies, a comparative analysis between constructs with different domain composition was performed to identify which constructs have the best-balanced size, stability, and functional rescue when it comes to sarcolemma localization and cytoskeletal linkage restoration [39]. These animal model studies have helped guide the optimization of vector design for translation into human trials.

Currently, micro dystrophin is the only approved gene therapy for DMD that is theoretically universal, assuming no pre-existing immunity to the AAV serotype used for delivery, as it holds no mutation-specificity (Table 1) [41]. However, the most profound limitation of this therapy is that it is not as effective in cardiac muscle due to poor AAV transduction, and cardiomyocarditis has been reported in 1 in 20 patients as a result of an immune response to the microdystrophin itself [43,44]. Microdystrophin therapy prevents heart failure in Fiona/dko mice but causes chronic cardiac inflammation in dKO mice despite functional rescue, highlighting a trade-off between efficacy and immune response [45,46]. These immune responses have led to a refinement of inclusion criteria in the various trails being undertaken. Pfizer now excludes patients with exon 9–13 or 29–30 deletions, while Sarepta excludes those with exon 1–17 or exon 45 deletions [41,47]. One of the microdystrophins, Elevidys from Sarepta, was approved by the FDA for use in ambulatory DMD patients aged 4–5 years in 2023. In 2024, this was extended to both ambulatory and non-ambulatory DMD patients four years and over.

**Table 1:** This table summarizes gene addition therapies for DMD.

Therapy	Status	Key Features	Clinical Outcome	Reference
SRP-9001/Delandistrogene moxeparvovec (Elevidys)	FDA approved: ambulant ≥4 y (traditional); non-ambulant ≥4 y (accelerated) US indication expanded June 20, 2024; Phase III ongoing	- Vector: AAVrh74 - Payload: Truncated micro-dystrophin - Promoter: MHCK7 (skeletal, cardiac, respiratory targeting) - Delivery and Dose: IV infusion, 1.33 × 10^14^ vg/kg over 1–2 h; pre-screen for anti-AAVrh74 TAb <1:400 - High cardiac expression profile - Manufacturing optimized for high capsid yield	- EMBARK 1 yr: +3.8 NSAA points vs. placebo (4–7 y) - ~40–50% of normal dystrophin expression on biopsy - CK reduction - Greatest gains in 4–5 y subgroup - EMBARK 2 yr: NSAA +2.88 vs. external control; TTR −2.06 s; 10MWR −1.36 s - Most common AEs: Vomiting, increased LFTs, decreased platelets - Mild acute liver injury in ~37% - Requires weekly LFTs ×3 mo, troponin-I ×1 mo, platelets ×2 wks; steroids ≥60 days - 2025: Temporary pause in non-ambulant shipments during FDA safety review	[48,49,50] NCT03769116 NCT05967351 NCT04626674 NCT05096221 NCT05881408 NCT06241950 NCT04547699 NCT03375164 NCT06128564 NCT06597656
RGX-202	Phase I/II ongoing 2022—ongoing	- Vector: AAV8 - Payload: Codon-optimized micro-dystrophin with C-terminal repeats retained - Promoter: Synthetic muscle-specific - Delivery and Dose: IV single dose; 1 × 10^14^ vg/kg and 2 × 10^14^ vg/kg cohorts - Large-scale suspension cell manufacturing for scalability	- Sustained muscle expression on biopsy (3–6 mo) - CK reduction - Well tolerated; mild AEs (vomiting, pyrexia) - Early evidence of muscle function benefit - Trial ongoing to evaluate long-term efficacy and safety	[51] NCT05693142 NCT03597581
SGT-003	Phase I/II 2023—ongoing	- Vector: AAV9 - Payload: Micro-dystrophin with nNOS binding domain - Promoter: Muscle-specific - Delivery and Dose: IV single dose; 1 × 10^14^ vg/kg and 2 × 10^14^ vg/kg under evaluation - Designed to restore nitric oxide signaling for improved perfusion	- Preclinical: Improved exercise tolerance vs. non-nNOS constructs - Early clinical: Safe at low dose, expression confirmed - Dose escalation ongoing - Designed to restore nitric oxide signaling to sarcolemma - Functional gains - Manageable safety events - IND-enabling studies support continued development	[52] NCT06138639
GNT0004	Phase I/II 2024—ongoing	- Vector: AAV8 - Payload: Micro-dystrophin (hMD1) - Promoter: Spc5.11 (skeletal and cardiac muscle) - Delivery and Dose: IV single dose; 1 × 10^13^ vg/kg (n = 2) or 3 × 10^13^ vg/kg (n = 3); sirolimus + steroids prophylaxis - Immune prophylaxis mitigates myositis risk	- Dose 2: mean 53% hMD1+ fibers, VCN 1.2, CK decreased 50–87% at week 16 - Early evidence of sarcolemma stabilization - Well tolerated after protocol amendments - 4 mild ADRs; SUSAR in first dose-1 patient - Robust preclinical efficacy in dystrophin-deficient models - Strong sarcolemmal targeting and expression - Trial temporarily paused due to SAE; resumed with protocol amendments	[53]
rAAVrh74. MCK.GALGT2	Phase I/II 2022—completed	- Vector: AAVrh74 - Payload: GALGT2 - Promoter: MCK (skeletal and cardiac muscle) - Delivery and Dose: Intravascular limb infusion (bilateral legs); 2.5 × 10^13^ vg/kg/leg and 5 × 10^13^ vg/kg/leg - Induces α-DG glycosylation; potential cardioprotection - Local delivery minimizes systemic exposure	- Higher-dose subject: NSAA 20→23, 6MWT +73 m at 24 mo - Glycosylation confirmed in both patients - No SAEs; ILI well tolerated - Modest improvements in strength - Slowed disease progression in subset of patients	[54] NCT03333590
FS344 (AAV1-Follistatin)	Phase I/II (BMD—completed) 2015—ongoing (for DMD)	- Vector: AAV1 - Payload: Follistatin (FS344→FS315) - Promoter: CMV - Delivery and Dose: Direct intramuscular (quadriceps); 3 × 10^11^ vg/kg or 6 × 10^11^ vg/kg per leg - Myostatin inhibition for hypertrophy; relevant to DMD muscle growth support	- BMD cohort results: Low dose (3 × 10^11^ vg/kg/leg): +58 m and +125 m in 6MWT High dose (6 × 10^11^ vg/kg/leg): +108 m and +29 m in 6MWT Histology: Decreased fibrosis, increased central nuclei, muscle fiber hypertrophy No gene transfer-related adverse events - DMD cohort: Primary endpoint: Safety Secondary endpoints: 6MWT, imaging, histology, immune monitoring, quality of life Results pending - Follistatin overexpression inhibits myostatin to promote muscle growth - Increased muscle volume - Enhanced strength and functional capacity - No serious adverse events reported	[55] NCT01519349 NCT02354781

## 4. Compensatory Gene Therapies for DMD

### 4.1. Utrophin

**Figure 4:**
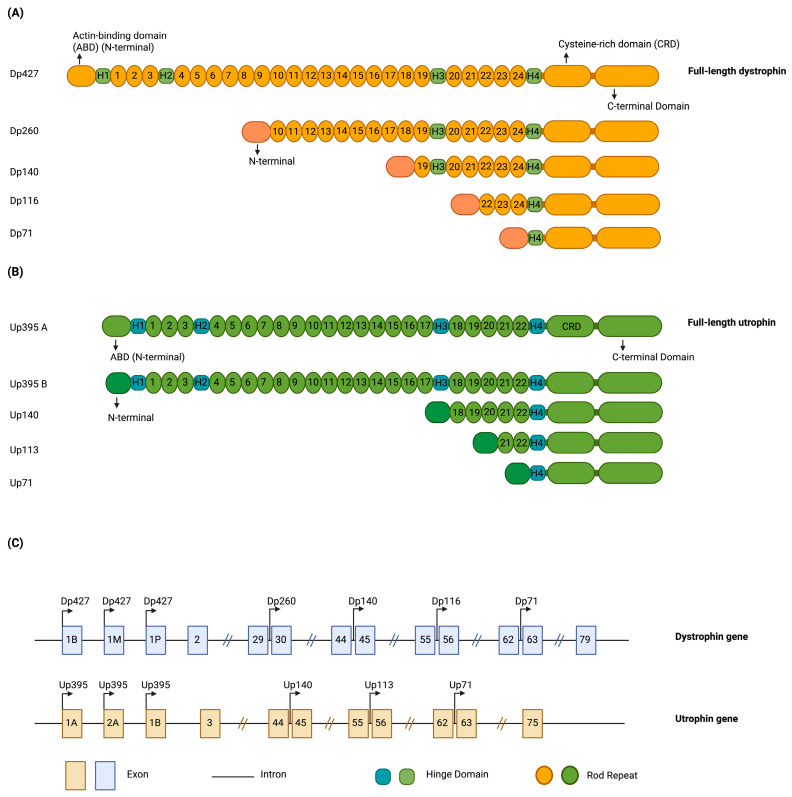
Comparison of dystrophin and utrophin genes and proteins. (**A**) Shows alternative transcripts of naturally produced dystrophin protein and their protein structures [85]. (**B**) Shows different transcripts of naturally occurring utrophin proteins and their protein structures [15]. (**C**) Shows the schematic comparison of *DMD* and *UTR* genes [15]. For dystrophin, the 1B, 1M, and 1P promoters all generate the full-length Dp427 isoform; although transcription can initiate at any of these sites, the alternative first exons splice into the common downstream exons to produce the same protein product. For utrophin, the 1A, 2A, and 1B promoters all give rise to the full-length Up395 isoform, while additional downstream promoters initiate shorter isoforms, such as Up113 and Up71. Isoform names such as Dp427, Dp260, and Up395 denote the approximate molecular weight (kDa) of the final protein product. “Created in BioRender. Rt, Z. (2025) https://BioRender.com/oxgx9uq (accessed on 11 October 2025).”

Utrophin has relevance for the treatment of DMD due to its structural and functional homology to dystrophin [57,58]. This similarity can be seen in their genes (80% homology) and protein sequences, as shown in Figure 4A,B [59]. Dystrophin (427 kDa, 3685 amino acids) and utrophin (395 kDa, 3433 amino acids) have some differences in their structural composition and specific roles [60]. Dystrophin’s central rod domain has 24 spectrin repeats and four hinges, linking to F-actin, β-dystroglycan, sarcoglycans, and laminin α2 to form the dystrophin-associated protein complex (DAPC), stabilizing the sarcolemma during muscle contraction and relaxation [60]. It recruits nNOS directly via spectrin repeats 16/17 and 20–23, and indirectly via syntrophins [60]. Utrophin, part of the utrophin-associated protein complex (UAPC), lacks spectrin-like repeats 15 and 19, binds actin only through its N-terminal domain, and cannot recruit nNOS directly, relying instead on syntrophin for this function [60]. Dystrophin is primarily expressed in skeletal and cardiac muscles, whereas utrophin is mainly found at neuromuscular junctions in adults [60]. Here, utrophin binds to Raspyn for acetylcholine receptor clustering, and its C-terminal domain binds to multiple asters (MAST), associating with microtubules and linking to laminins α4, α5, and β2 [60]. The developmental expression of utrophin and dystrophin differs between species. In mice, skeletal muscle fibers predominantly express utrophin at birth, which is gradually replaced by dystrophin postnatally. In humans, however, utrophin expression in skeletal muscle is largely confined to fetal stages, where it peaks around 17–18 weeks of gestation, declines sharply by 26 weeks, and is virtually absent at term birth, except at the neuromuscular junction or sub-sarcolemmal regions [61]. Although, not completely replicating dystrophin’s form and function, it is hypothesized that because newborn mouse muscle and fetal human muscle express utrophin instead of dystrophin, the upregulation or gene transfer of utrophin could be beneficial for DMD patients [58].

This is supported by recent findings showing that reduced sarcolemmal utrophin correlates with more severe clinical phenotypes in DMD patients, and that the failure of utrophin to localize to the membrane may underlie unusually severe disease in cases with in-frame dystrophin deletions [62,63]. Furthermore, pharmacological screening has identified 2-pyrimidine carbohydrazides as potent small molecules capable of upregulating utrophin, representing a promising genotype-independent therapeutic strategy [64]. An upregulation therapy which entered clinical trials was the small molecule SMT C1100 [58]. In *mdx* mice, SMT C1100 produced a two-fold upregulation of utrophin and significantly improved dystrophic muscle pathology by reducing fibrosis, inflammation, and central nucleation, while enhancing membrane stability and muscle function, leading to clinical trial testing [65]. In Phase 2 clinical trials, SMT C1100 was administered orally at 100 mg/kg twice daily for up to 48 weeks which successfully upregulated utrophin production; patients, however, did not see any improvements due to the rapid clearance of the drug [66,67]. Thus, the SMT C1100 clinical trial was discontinued. Although SMT C1100 has been discontinued, recent studies have identified several promising small molecules that upregulate utrophin and similarly to SMT C1100 have reduced fibrosis in *mdx* mice [68,69]. Quinazoline and quinoline scaffolds showed nanomolar potency via AhR antagonism, while 3D-QSAR and docking studies of 2-pyrimidine carbohydrazides revealed key features for transcriptional activation [70,71]. Second-generation phosphinate esters also improved pharmacokinetics and efficacy over earlier compounds [72]. Taken together, recent progress points to an expanding set of utrophin-targeting compounds that may hold real promise for therapeutic use.

Additionally, utrophin can be upregulated via transcriptional factors. For example, artificial zinc-finger transcriptional factors (ZFP-ATFs), such as Jazz and JZif1, have been designed to bind to utrophin’s A promoter at its CG-rich region and cause activation. Jazz (a three-finger ZFP fused to Vp16 activation domain) delivered via AAV8 increased utrophin mRNA and protein levels in *mdx* mice, improving muscle function and resistance to contraction-induced injury [73,74]. This led to development of an evolved version of the artificial zinc-finger activator called JZif1, which led to two times stronger upregulation of utrophin. JZif1 also showed better transduction in *mdx* due to the use of muscle AAV (mAAV), a modified version of AAV8 [75]. These results demonstrate that harnessing engineered transcriptional regulatory machinery offers a powerful strategy for increasing utrophin levels to treat DMD genotypes regardless of dystrophin mutation status.

Other strategies are currently under in vitro development, including the use of Cas9-directed cleavage to disrupt the downregulating microRNAs, such as miR-150, miR-296-5p, miR-133b, let-7c, and miR-196b, leading to two-fold upregulation [76]. This has not yet been tested in animal models, and there is the potential for off-target double strand break (DSB) formation using catalytically active Cas9. However, non-editing strategies have been used to upregulate utrophin in *mdx* mice by blocking microRNA repression. A miR-206 decoy, delivered via AAV9, increased utrophin expression by 2.5-fold and improved muscle histology [77]. Similarly, a PMO-based site-blocking oligonucleotide (SBO) targeting the let-7c binding site on the utrophin 3′UTR, where let-7c miRNA normally binds to repress utrophin translation, was administered by intramuscular injection to *mdx*. SBO targeting resulted in a 2-fold increase in utrophin protein expression and reduced muscle degeneration. These mutation-independent approaches show promise for DMD therapy [78].

Similar to micro-dystrophin, AAV-delivered micro-utrophin has also been developed but it has not yet reached human trials [79]. Micro-utrophin therapy showed a consistent and similar efficacy to micro-dystrophin therapy in animal studies [79]. In fact, micro-utrophin therapy induced a much lower cellular immune response relative to micro-dystrophin [79]. Codon-optimized human micro-utrophin constructs driven by MHCK7 and SPc5-12 (muscle-specific promoters) were delivered to both *mdx* and D2/*mdx* mice via AAV9 which showed improvements in skeletal and cardiac muscle function. Additionally, normalized serum creatine kinase, a favorable safety profile at high vector doses and minimal off-target expression or inflammatory response in wild-type rats were observed [80,81]. In the GRMD dog model, micro-utrophin reduced histological signs of damage and elicited negligible T-cell responses in contrast to micro-dystrophin [82]. Notably, in the *mdx*4cv mouse model, micro-utrophin preferentially restored integrity to fast-twitch type IIb fibers and improved the neuromuscular junction [83]. Furthermore, earlier studies using alternative vector configurations also demonstrated reduced inflammation, successful sarcolemma localization of micro-utrophin, and increased lifespan in dystrophin/utrophin-deficient mice treated with AAV6 micro-utrophin [84]. These findings highlight micro-utrophin’s potential as a universal, mutation-independent therapeutic candidate with a significantly improved immunological profile compared to micro-dystrophin.

### 4.2. Follistatin

**Figure 5:**
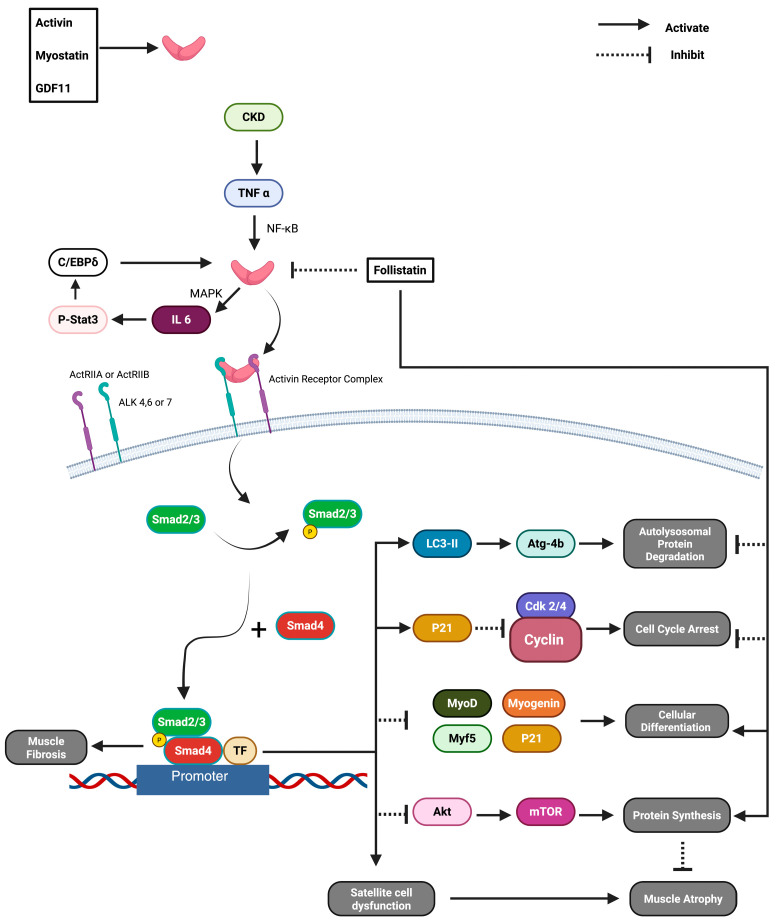
Myostatin signaling pathway. This figure shows how myostatin binding with the activin–receptor complex will lead to cell cycle arrest, protein degradation, and the halting of muscle growth. It also shows that follistatin is an antagonist to myostatin which reverses these effects [92]. “Created in BioRender. Rt, Z. (2025) https://BioRender.com/6izxs7h (accessed on 11 October 2025).”

Follistatin is a glycoprotein that is expressed in most tissues [86]. Follistatin has many roles in the body, such as muscle growth regulation and follicle-stimulating hormone (FSH) release regulation [87]. Follistatin inhibits the myostatin/activin pathway, as shown in Figure 5, and thereby promotes muscle growth and the inhibition of fibrosis [87]. An AAV1 CMV-driven follistatin gene transfer therapy has been developed and tested in Phase l/ll clinical testing in DMD’s less severe allelic disease Becker MD (BMD) (Table 1) [87]. The treatment was administered to BMD patients aged 24–37 years. Muscle biopsies were taken pre-treatment and at 30 days and 6 months post-treatment. Additionally, patients underwent a six-minute walking test pre-treatment, and at 30, 60, 90 days, and six months post-treatment. Both assessments showed improvement in their BMD disease conditions. Muscle mass and muscle force generation also improved, and fibrosis was reversed in these patients [55,87,88]. For BMD, where a truncated semi-functional dystrophin is present, follistatin’s ability to block fibrosis and atrophy is beneficial. Even though this may not be replicated in DMD due to the complete absence of dystrophin expression, follistatin gene therapy for DMD patients is currently in its Phase l/ll trial.

Further studies have reinforced follistatin’s therapeutic potential through protein engineering and pharmacological modeling. A quantitative systems pharmacology (QSP) model was developed to investigate the efficiency of FS-EEE-Fc, which is a follistatin recombinant protein for DMD treatment. QSP predicted that weekly doses of 3–5 mg/kg could achieve a 7–10% increase in muscle volume through the dual inhibition of myostatin and activin pathways. This model was parameterized using preclinical data from C57BL/6 wild-type and myostatin-overexpressing mice and included efficacy trends observed after dosing in *mdx* mice, thus providing a more disease-relevant basis for dose prediction [89]. In parallel, the development of ACE-083 which is a locally acting follistatin–ligand trap fusion protein, promises therapeutic potential by neutralizing myostatin, activin A, activin B, and growth differentiation factor 11 (GDF11). ACE-083 has shown localized muscle hypertrophy in C57BL/6 wild-type, Trembler-J (Charcot-Marie-Tooth disease model), and *mdx* mice, where intramuscular administration into the tibialis anterior muscle increased muscle mass by approximately 77% in *mdx* and 116% in wild-type mice, and boosted absolute isometric force generation [90]. Another recent protein-engineering study further validated follistatin as a flexible scaffold for recombinant constructs, demonstrating enhanced ligand binding and pharmacokinetics with in vivo efficacy tested in C57BL/6 mice. In this study, researchers employed rational mutagenesis and fusion strategies to enhance the stability, receptor binding affinity, and serum half-life of follistatin. They introduced three point mutations K76E, K81E, and K82E in the heparin-binding loop of follistatin-315 to eliminate heparan–sulfate binding. This modification, known as FST-ΔHBS-Fc, significantly extended the protein’s half-life and systemic exposure while maintaining its ability to inhibit myostatin and activin A. In C57BL/6 mice, this engineered version led to increased muscle mass and enhanced regeneration following injury [91]. The results seen with these follistatin-based constructs in *mdx* mouse models support their potential for clinical translation, especially in the context of combination therapies aimed at improving both muscle regeneration and reducing fibrosis in DMD.

### 4.3. β1,4-N-Acetylgalactosaminyltransferase-2

The *GALGT2* or *B4GALNT2* gene expresses a protein called β1,4-N-acetylgalactosaminyltransferase-2 which catalyzes the glycosylation of proteins. In muscle, this would include α-dystroglycan. In the absence of dystrophin, this has the potential to protect the sarcolemma from contraction-induced damage. It will also act to upregulate the expression of several genes including those encoding utrophin, laminin α4, α5, and integrin [93,94,95,96,97]. These downstream effectors of GALGT2 are highly localized to the neuromuscular junction and myotendinous junction in adult skeletal muscle and have a relatively low expression in the heart. The potential therapeutic capacity of GALGT2 has been studied and established in several mouse models of muscular dystrophies, including *mdx* mice, which is one of the most common animal models of DMD. These studies show that the AAV gene addition of GALGT2 prevents the loss of cardiac function in the aging *mdx* mouse heart as well as preventing the eccentric contraction induced damage to the skeletal muscles [98]. In *mdx* mice, deletion of *Galgt2* worsened both skeletal and cardiac pathology, which led to increased inflammatory infiltration and impaired cardiac function [99]. Conversely, the overexpression of GALGT2 in skeletal muscle induced glycosylation changes that enhanced α-dystroglycan function and significantly reduced contraction-induced injury in both *mdx* and wild-type muscle fibers, which indicated GALGT2 acted to stabilize the sarcolemma independent of dystrophin [100]. Importantly, the transgenic overexpression of *Galgt2* in utrophin-KO *mdx* mice protected against dystrophic pathology, which indicates that GALGT2’s beneficial effects can also occur independently of utrophin upregulation [101]. Furthermore, studies in large-animal models support the therapeutic potential of GALGT2. In GRMD dogs treated with rAAVrh74.MHCK7.GALGT2, a widespread increase in α-dystroglycan glycosylation and upregulation of utrophin in both skeletal and cardiac muscle was observed, but only modest histological improvements were seen and no significant gains in muscle strength were reported over the 3-month study period [102]. These findings reinforce the potential of GALGT2 as a mutation-independent therapeutic agent but highlight the need for early intervention and possible combination strategies to enhance functional outcomes.

In 2022, a Phase I/II clinical trial using AAVrh74 to deliver *GALGT2* cDNA driven by a MCK promoter was performed in two ambulant DMD patients at 6.9 and 8.9 years old at 1 × 10^14^ vg/kg and 5 × 10^13^ vg/kg total dose (Table 1) [54]. One of the patients showed a meaningful biological response, including evidence of GALGT2 expression and an initial modest upregulation of downstream targets such as utrophin and α-dystroglycan glycosylation. There were no adverse effects, and the treatment was well tolerated. The rationale behind selecting younger patients and exploring higher vector doses was based on two preclinical studies conducted prior to the clinical trial which demonstrated that younger *mdx* mice exhibited stronger biological responses to GALGT2 gene transfer, and that higher vector doses led to greater expression and downstream activation of protective proteins such as utrophin and laminins [98,103]. GALGT2 cDNA has also been delivered using non-viral vectors in *mdx* mice. This approach led to strong GALGT2 expression in the liver while expression in skeletal and cardiac muscle was minimal, and no functional improvements were reported. These findings highlight a major challenge with current non-viral vector systems in DMD as they struggle to effectively target muscle tissue. This reinforces the need to develop delivery platforms specifically designed for skeletal and cardiac muscle to improve therapeutic outcomes [104].

### 4.4. Klotho

Klotho, encoded by the *KL* gene, is recognized as an anti-aging gene [105]. Mutations in the *KL* gene lead to significantly shortened lifespans and pronounced accelerated aging phenotypes in mice, manifesting as conditions such as arteriosclerosis, osteoporosis, and skin atrophy. The overexpression of Klotho has been demonstrated to increase the lifespan of mice, suggesting its potential role in longevity [106]. Moreover, Klotho exhibits notable anticancer properties by inhibiting cancer cell proliferation, migration, and invasion [107]. The diverse properties of Klotho are due to its involvement in multiple crucial signaling pathways, including the insulin/IGF-1 pathway, which is essential for metabolic regulation; the Wnt signaling pathway, which is critical for cell proliferation and differentiation; the p53/p21 pathway, which is involved in cell cycle regulation and apoptosis; the cAMP pathway, which influences cellular responses to hormonal signals; protein kinase C, which is involved in regulating various cellular functions; and the TGF-β pathway, which plays a role in cellular growth and differentiation [108]. In the *mdx* mouse model of DMD, chronic inflammation, oxidative stress, epigenetic modifications, and repetitive muscle damage collectively act to silence the *Kl* gene, exacerbating disease pathology [109]. Building on these findings, a study showed that a 50% increase in muscle expression of α-Klotho in *mdx* mice led to a 50% increase in forelimb grip strength relative to non-transgenic *mdx*4cv littermates, despite no measurable improvement in voluntary wheel running behavior [110]. This indicates that while α-Klotho can enhance muscle strength, it does not fully restore endurance or mitigate the systemic features of dystrophic pathology. It is worth mentioning that recent studies have further defined the role of Klotho in DMD pathology. In a study, *mdx* mice with muscle-specific overexpression of a Kl transgene exhibited preserved cardiac function by preventing left ventricular ejection fraction and fractional shortening deficits. Histological analyses revealed reduced cardiac fibrosis and lowered the expression of oxidative damage markers while reducing expression of TGF-β and fibrotic genes such as fibroblast growth factor 23 (FGF23) [111]. Together, these studies show the importance of KL as a regulatory factor; its downregulation in dystrophic muscle contributes to disease progression and increased fibrotic remodeling while showing KLs therapeutic potentials for treating DMD.

## 5. Concerns Surrounding Gene Therapy Approvals for DMD

The approvals of each of the therapies described above have been controversial. The levels of dystrophin protein seen with exon skipping and Translarna are well below the 20% suggested to be required for therapeutic benefit. The phenotypic improvements seen in Phase 3 trials of Elevidys were marginal and secondary outcomes were not met. Clinical trials in DMD are complicated by variation in disease progression between patients, and disparity in outcome improvements dependent on the stage of disease at the commencement of treatment. This has been improved more recently with natural history studies. Very recently, the liver failure related deaths of three patients on AAV therapies developed by Sarepta (two of which were treated with Elevidys) has led the FDA to request the company voluntarily stop all shipments of the drug [50,112]. This will allow time for the safety labeling supplement process to be implemented. It should be noted that the deaths were in older non-ambulatory patients; the safety in younger ambulatory patients is better, but there is still an acknowledged risk of death from liver, kidney, heart, or lung failure with high dose of systemic AAV treatments [113]. The toxicity is driven by the vector-directed innate and adaptive immune responses, with the respiratory insufficiency and cardiomyopathy seen in DMD patients also contributing to patient death. Current strategies to remove the risk of immune-related toxicities include screening patients for pre-existing AAV antibodies and specific AAV capsid T cells, and the use of immune-modulating drugs before, during, and after treatment. However, our understanding of the mechanisms that lead to the immunotoxicities seen with high doses of AAVs is limited, and as no standardized management protocols have been established yet.

Much work is now focused on the development of engineered capsids to improve transduction efficiency so that lower doses could be used and provide more tissue-specific targeting to reduce liver toxicities. The engineered myotropic vectors, AAVMYO and MyoAAV, that have capsids bearing the amino acid motif RGD (arginine–glycine–aspartate), display the highest muscle transduction rate in small and large animal models thus far reported [114]. Engineering of the vector genome itself to (i) remove CpG motifs, recognized by the immune system as a sign of microbiological presence; (ii) include inhibitory oligos that antagonize Toll-like receptor 9 (TLR9) activation; and (iii) include microRNA binding sites to facilitate endogenous miRNA-mediated regulation to de-target transgene expression away from antigen presenting cells to reduce transgene immunity, is also being explored [115,116]. Others are working on the development of electrostatically coated vectors, artificially enveloped AAV vectors, and extracellular vesicle-encapsulation of AAV vectors to prevent immune recognition [117,118,119].

## 6. Combination Therapies

There is a growing consensus that combination therapies will be necessary to effectively manage the complex pathology of DMD. Regulators and trial protocols customarily require that patients must have been on a stable dose of corticosteroids for at least 12 weeks before inclusion on a gene therapy or exon skip trial. Corticosteroids act to reduce the inflammation that results from muscle damage. Reducing the atrophic and fibrotic phenotypes could potentially improve the efficacy of the gene therapies by preserving the musculature [120]. The AAV gene therapies described above that would compensate for the lack of dystrophin (microdystrophin or microutrophin), or act to address the phenotype (follistatin or GALGT2) could theoretically be used in combination. Because of the limiting packaging capacity of the AAV vectors, this would require treatment with two AAV vectors which would be associated with high risks. A recent study demonstrated that dual AAV delivery of mini-dystrophin and VEGF synergistically improved muscle pathology in *mdx*’s temporalis muscle. Co-treatment enhanced dystrophin-associated protein complex formation, increased angiogenesis, and significantly reduced inflammation and fibrosis compared to monotherapies [121]. Molecules have been used to upregulate utrophin expression, and recombinant follistatin protein has also been trialed in mouse models; these could replace the need for two AAV vector combinations but a lot of optimization of dosing regimens, in all likelihood to suit the stage of disease progression, would be required. An alternative strategy to upregulate gene expression could be achieved with CRISPRa systems.

## 7. CRISPRa Systems

**Figure 6:**
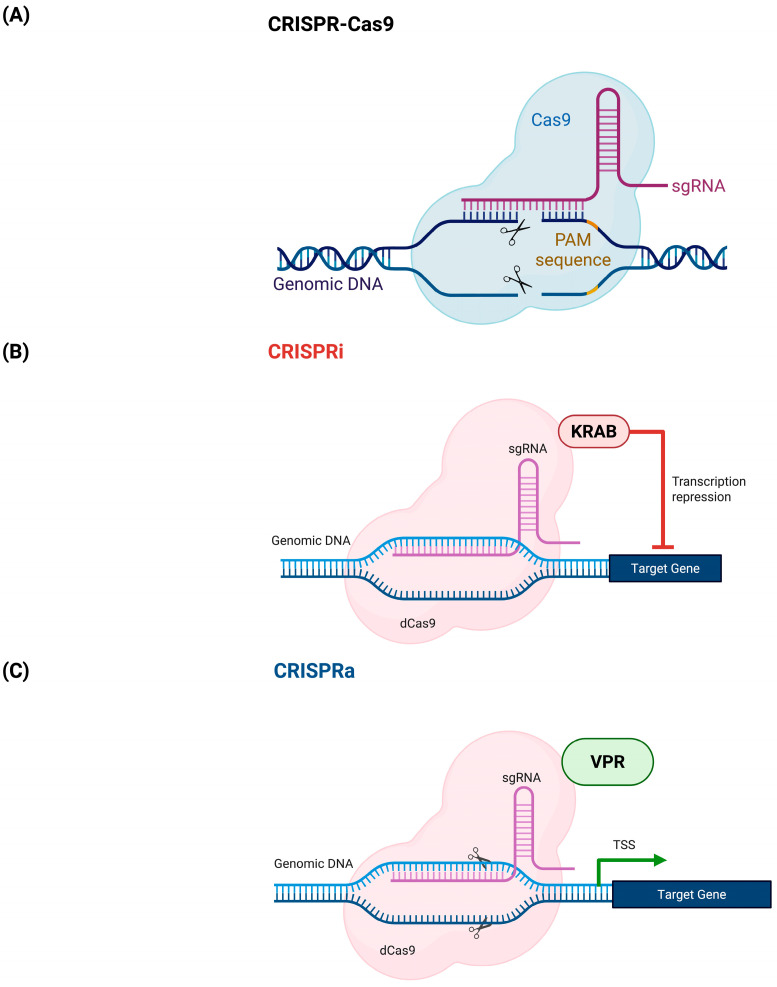
Different CRISPR systems. (**A**) Shows a traditional catalytically active CRISPR-Cas9 system with the capacity to make a DSB. (**B**) Shows a CRISPRi system where catalytically deactivated Cas9 is linked to a transcriptional suppressor. (**C**) Shows a CRISPRa system where catalytically deactivated Cas9 is linked to a transcriptional activator. “Created with BioRender.com with part a: adapted from “CRISPR-Cas9 Gene Editing in Trypanosoma cruzi”, by BioRender.com (2024) and retrieved from https://app.biorender.com/biorender-templates (accessed on 15 June 2024); part b: reprinted from “dCas9-KRAB Transcriptional Repression System”, by BioRender.com (2024) and retrieved from https://app.biorender.com/biorender-templates (accessed on 15 June 2024); and part c: a adapted from “CRISPR-mediated Transcriptional Activation”, by BioRender.com (2024) and retrieved from https://app.biorender.com/biorender-templates (accessed on 15 June 2024). Created in BioRender. Rt, Z. (2025) https://BioRender.com/x95abji (accessed on 11 October 2025)”.

Clustered Regularly Interspaced Short Palindromic Repeats (CRISPR)-Cas9 is the bacterial immune system against bacteriophages [44]. CRISPR-Cas9 has the capacity to be programmed for eukaryotic genome editing using single stranded guide RNA (sgRNA)- complementary to the target DNA sequence bearing a relevant protospacer adjacent motif (PAM) that acts to direct the Cas9 to make a double stranded break (DSB) at a desired site, as shown in Figure 6A [122]. This targetability of Cas9 has made it a great gene editing tool in recent years. Cas9 has also been re-engineered to enable its use to upregulate (CRISPRa) or downregulate (CRISPRi) genes of interest instead of creating a DSB in the DNA (Figure 6B) [123]. In CRISPRa, Cas9 is deactivated by point mutations at the nuclease domains of the enzyme to create dead Cas9 (dCas9) [124]. Then, dCas9 is linked to a transcriptional activator such as Vp64-p65-Rta (VPR) or P300 (a histone modifier) and the sgRNA is designed to target −400 to −50 bases from the transcriptional start site (TSS) of the gene of interest, as illustrated in Figure 6C [125,126].

### 7.1. Design and Evolution of CRISPRa

CRISPRa technology developed rapidly following the foundational studies on CRISPR-Cas9, with the first activation methods emerging in 2013–2014. These early systems commonly relied on a shared framework, which is the fusion of catalytically inactive Cas9 (dCas9) with one or more copies of the herpes simplex virus transcriptional activation domain (TAD), VP16. This domain is also referred to as Vmw65 or a-TIF, and its name often changes with the number of repeats. For example, four tandem repeats of VP16 are termed VP64. The minimal TAD region required for activity comprises 10 amino acids [127,128,129,130,131]. Although these CRISPRa systems were functional, their transcriptional induction was modest, typically not exceeding tenfold activation of endogenous genes, even when incorporating up to ten copies of VP16.

The level of activation needed for a particular target also varies. In some cases, a small increase (a two- or three-fold) is enough to cause a biological effect, while in others, a higher-fold change in expression may be required. Typically, it is easier to achieve an increase in expression at a much higher fold if a gene is not expressing at all, compared to achieving an increase in expression for a target which is already expressed in a cell type [132]. Also, the level of expression varies across different cell types for the same target. Recently, a comprehensive review summarized the current and emerging activation domains employed in CRISPR activation systems, elucidating how single and combinatorial domain architectures modulate transcriptional potency, target specificity, and robustness of CRISPRa screens across diverse applications [133].

To facilitate CRISPRa in eukaryotic cells, catalytically inactive Cas9 (dCas9) has been fused to transcriptional activators, such as VP16 repeats specifically VP64 (four copies), VP160 (ten copies), and VP192 (sixteen copies) as well as the p65 activation domain (p65AD). These dCas9-fusion proteins, including dCas9-VP64 and dCas9-p65AD, have consistently activated the expression of various reporter and endogenous genes to moderate significant levels in *Saccharomyces cerevisiae*, mouse, and human cells, with high specificity [129,130,134,135,136]. The dCas9-VP160 construct has been used to upregulate *Laminin Subunit Alpha-1* in C2C12 cells and mouse muscle following plasmid electroporation, and in a screen targeting estrogen-responsive genes [137,138]. Meanwhile, dCas9-VP192 has been shown to effectively induce pluripotency genes across various human cell types [139].

### 7.2. Multiple Effector Domains for CRISPRa

Along with increasing the number of VP16 domains, in order to boost gene expression, combinations of activation domains are often used. In one such system, dCas9 is fused to a SunTag array comprising 10 tandem copies of a small peptide epitope designed to recruit multiple molecules of an antibody-fusion protein containing a cognate single-chain variable fragment (scFv), superfolder GFP (sfGFP), and the VP64 activation domain. This dCas9–SunTag–VP64 configuration significantly enhances transcriptional activation by amplifying the recruitment of activator domains, leading to strong and specific upregulation of endogenous human genes using single sgRNAs, surpassing the activation levels achieved by dCas9–VP64 alone. Moreover, the system has been shown to both activate poorly expressed genes and further elevate expression of already well-expressed genes [128,140].

The SAM system enhances the basic dCas9-VP64 setup by adding p65 and HSF1 activators, recruited through MS2 stem-loops in a modified sgRNA. These loops bind to MS2 coat protein (MCP), which is fused to the activators. In a 2016 study, VPR, Suntag, and SAM all outperformed VP64 alone. Gene activation varied ranging from 10-fold to 10,000-fold depending on the gene, guide RNA, and cell type. New CRISPRa systems continue to evolve by combining existing tools. For example, dCas9-VPR fused with p300 (dCas9-VPRP) enhances gene activation beyond what either component achieves alone [141]. VPR has also been paired with miRNA sensors to recruit more effectors, resulting in up to six-fold higher activation. Other advanced systems include SPH (SunTag-p65-HSF1), which performs better than SAM or SunTag alone, and TREE, which integrates SAM and SunTag elements and doubles VPR’s activation. TREE uses MS2-MCP to tether 16 copies of an activator mix (GCN4, MCP, scFv, and p65) [142].

**Figure 7:**
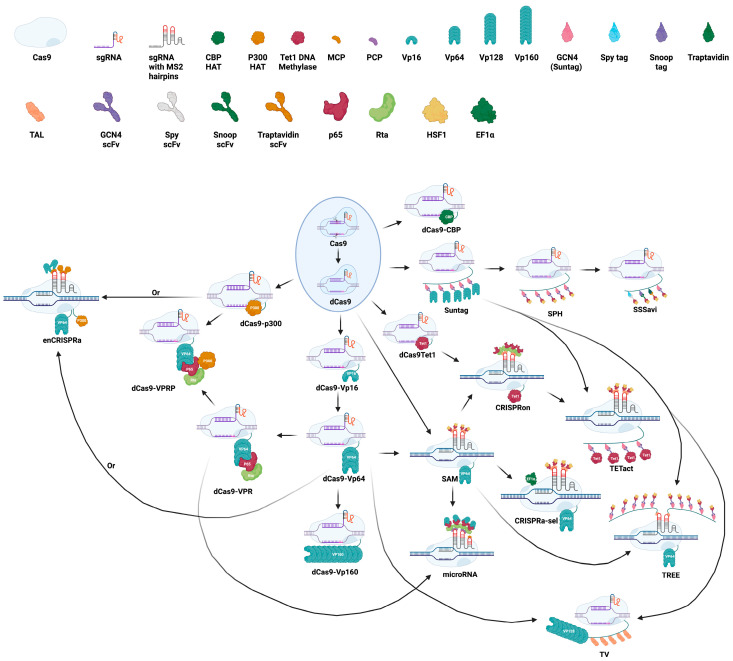
Evolution of CRISPR-mediated transcriptional activation systems. The schematic traces the development of CRISPRa platforms from early designs using a single activation domain, such as VP16, VP64, VP160, and VPR directly fused to dCas9 through intermediate strategies incorporating stronger or multiple activators to advanced multi-component architectures. Later generations, such as SunTag, SAM, SPH, and TREE, employ protein or RNA-based scaffolds to recruit combinations of transcriptional chromatin epigenetic modifiers (e.g., p300, CBP) and DNA epigenetic regulators (e.g., Tet1). “Created in BioRender. Rt, Z. (2025) https://BioRender.com/nyk9wrr (accessed on 11 October 2025)”.

Through a comparative screen of dCas9 fused to over 20 distinct activation domains to identify constructs capable of robust reporter gene induction, a tripartite activator comprising a tandem fusion of VP64, the NF-κB p65 activation domain (p65AD), and the Epstein–Barr virus R transactivator (Rta), collectively referred to as VPR, was engineered and characterized. The dCas9–VPR fusion markedly outperformed dCas9-VP64 in activating endogenous transcription in *Saccharomyces cerevisiae*, *Drosophila melanogaster*, and both murine and human cell lines. Furthermore, dCas9–VPR enabled robust, multiplexed activation of multiple genes simultaneously. In a subsequent study directly comparing the SunTag, SAM, and VPR systems, it was found that while SAM demonstrated the most consistent gene activation across multiple targets, the relative performance of each system was context-dependent, with SunTag and VPR occasionally outperforming SAM depending on the target gene. Another recent development is the SSSavi system, which improves gene activation by combining four activation domains such as SpyTag, SnoopTag, SunTag, and AviTag. The system is highly modular, and gene activation varied depending on the order and number of domains used (Figure 7) [143].

Development continues with innovative designs such as the three-component repurposed technology for enhanced expression (TREE) system. The TREE combines dCas9-VP64, engineered sgRNAs exposing hairpins, and MS2-fused epitope tags recognized by ScFv antibodies fused to p65 and HSF1. This large, multicomponent system reminiscent of a tree in structure outperforms some SAM, VPR, and SunTag variants in transcriptional activation potency [144]. Parallel efforts are underway to improve plant-specific CRISPRa systems, which may yield insights applicable to mammalian applications in the future [145,146]. Several CRISPRa systems have also been modified to allow the precise spatial and temporal control of gene activation. One approach utilizes optogenetics, where dCas9-VP64 is coupled with blue light-responsive proteins CRY2 and CIB1 to control transcription with light exposure [147]. Gene activation using CRISPRa depends on several factors, including cell type, chromatin structure, and the baseline expression of the target gene.

There have been approaches to use CRISPRa as a therapeutic approach to treat different diseases including genetic disorders, retinal diseases, metabolic disorders, and various cancers [148]. CRISPRa has been used to treat the most common inherited type of blindness known as retinitis pigmentosa in a rhodopsin-deficient mouse model via activation of the *Opn1mw* (M-opsin) gene in rod photoreceptors [149]. In another study, the activation of the pancreatic and duodenal homeobox gene 1 (*Pdx1*) in liver cells led to the formation of insulin-producing cells, presenting a possible new therapy for type I diabetes [150]. In a recent study, a comparable CRISPRa technique was utilized to simultaneously activate multiple endogenous genes such as *Cd70*, *Cd80, Cd86*, *Ifnα4*, *Ifnβ1*, and *Ifnγ*, triggering anti-tumor adaptive immune clearance of tumor cells in a mouse model [151].

## 8. Application of CRISPRa to DMD

### 8.1. Dystrophin Isoforms Other than Dp427m

For mutations that allow it, the upregulation of different isoforms of dystrophin is possible with CRISPRa. In a case study, a 27-year-old patient had a mutation in the promoter 1M (Figure 4C), which regulates the production of the muscle dystrophin isoform of the *DMD* gene [152]. He was treated with a d*Sa*Cas9-Vp64 targeting promoter B (Figure 4C) of the *DMD* gene, that drives brain dystrophin isoform expression, delivered via an AAV9 vector [152]. Promoter B was targeted because it is intact and can be artificially activated to bypass the defective muscle promoter, still producing full-length dystrophin. However, the patient at 27 years of age had advanced DMD and died of acute respiratory distress syndrome (ARDS) because of their innate immune reaction to the high dose of AAV9 used [152]. This case underscores how risky AAV gene therapies can be, regardless of whether they are used for gene replacement or upregulation, and emphasizes the importance of treating patients earlier in the disease course, when pathology is less advanced. Nonetheless, risk with systemic AAV vectors is principally dose-dependent; serious adverse events, including fatal immunotoxicity, have been associated with high vector loads, particularly in patients with underlying hepatic, cardiac, or renal compromise [113]. Accordingly, establishing an indication- and patient-specific therapeutic window remains essential as in practice, lower systemic doses may be better tolerated yet yield modest clinical effect (e.g., delandistrogene moxeparvovec), whereas higher exposure can improve expression at the expense of safety in certain settings (e.g., onasemnogene abeparvovec) [48,49,153,154,155].

### 8.2. Other Examined Targets

CRISPRa has been used to upregulate several genes that have therapeutic potential for DMD treatment. An in vivo study of follistatin activation in Cas9-expressing *mdx* mice demonstrated notable antifibrotic effects and significant improvements in both fore–limb and hind–limb grip strength, underscoring the potential of myostatin inhibition in ameliorating dystrophic muscle pathology via follistatin CRISPRa activation [150]. Building on the fact that follistatin gene transfer Phase l/ll in humans have been effective in patients with BMD with residual dystrophin function, follistatin gene upregulation using CRISPRa could, with much development, be used for DMD treatment and complement the current dystrophin restorative therapies. In the same *mdx* model, Klotho upregulation via CRISPRa led to increased muscle mass and further enhanced grip strength, suggesting that an increase in Klotho expression could complement dystrophin-restorative approaches [150]. Further studies using human neuronal and renal cell lines demonstrated that the dCas9–SAM system can effectively boost Klotho expression, reinforcing its potential as a candidate for gene activation therapy [156].

GALGT2 has also been targeted using dSpCas9 conjugated to a gene activator that combined VP64, p65, and HSF1 with a SWI/SNF chromatin remodeling complex. This was delivered as mRNA alongside designed and in vitro validated sgRNAs packaged into lipid nanoparticles (LNPs) and administered systemically by tail vein injection into C57BL/6J wildtype mice. This gene activator led to a dose-dependent enhancement of expression of GALGT2 that was 100-fold higher than that seen with VPR and p300. There was a peak in expression at 24 h post-injection, which decayed to baseline after 12 days. Re-administration of the loaded LNPs gave a similar peak and decay curve in expression. These findings highlight the potential of the gene activator and LNP platform to support repeat dosing, which is a critical feature for sustained therapeutic benefit [104]. Although the expression was examined in the liver, with adaptation of the LNPs with ligands for targeting of muscle, this work holds potential for DMD.

CRISPRa-based gene activation has also been used to activate utrophin in Cas9-expressing *mdx* mice which have a mutation in the dystrophin gene, and were genetically modified to express Cas9, enabling targeted gene activation upon sgRNA delivery. Initial AAV-delivered sgRNA in Cas9-expressing *mdx* mice achieved significant utrophin upregulation and led to the functional rescue of dystrophin [150]. Building on this foundation, single AAV vectors engineered to target muscle (MyoAAV-UA) carrying a compact dCasMINI-VPR activator was used to activate endogenous full-length utrophin across multiple DMD models including patient-derived iPSC-myotubes and in non-human primates which led to increased strength without causing toxicity [157]. In parallel, recent work in *mdx* identified a downstream enhancer of the utrophin gene (DUE) that is epigenetically repressed by the histone deacetylase SIRT6. In *mdx* mice, the genetic inactivation of SIRT6 led to increased acetylation of H3K56 at the DUE, resulting in robust utrophin upregulation and improved muscle function. Furthermore, CRISPR-dCas9 tethering of SIRT6 to the enhancer was sufficient to repress utrophin expression, confirming that the DUE acts as a critical locus for regulatory control [158]. These results suggest that CRISPRa could potentially be used not just at gene promoters but also at enhancer regions like the DUE through epigenetic activation. Targeting these regulatory elements might allow for stronger or longer-lasting gene activation and creates new possibilities for combining gene activation with existing dystrophin-restoring therapies in DMD.

## 9. Does CRISPRa Hold Potential for DMD?

**Figure 8:**
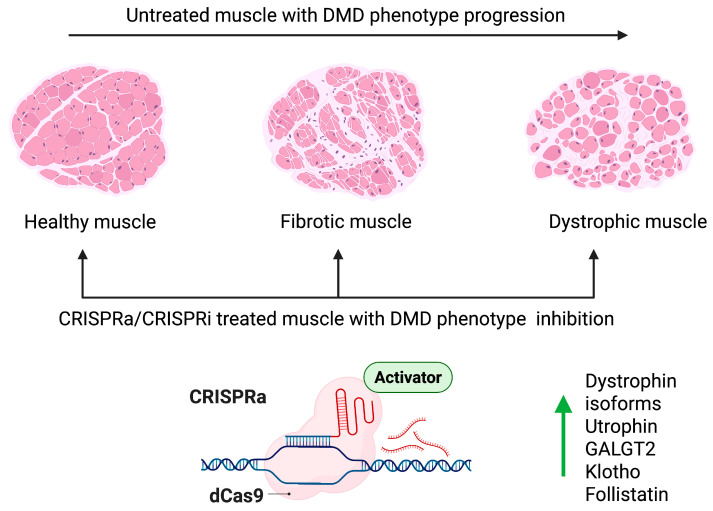
Dystrophic muscle phenotype progression and potential impact of CRISPRa on muscle phenotypes. This figure shows difference between muscles with DMD and healthy muscle. It also shows how the DMD muscle biopsy phenotype progresses. It also illustrates how CRISPRa upregulation of compensatory genes can intervene and halt the progression of DMD. “Created in BioRender. Rostamitehrani, Z. (2025) https://BioRender.com/ihnwqjq (accessed on 11 October 2025)”.

The ability to multiplex CRISPRa provides a potentially advantageous approach for treating DMD. However, the timing of the treatment and degree of upregulation would need to be tailored to the specific targets involved. Early interventions could help prevent or minimize muscle damage and fibrosis, whereas later interventions might focus on enhancing muscle regeneration and function (Figure 8). For different targets such as upregulating utrophin and upregulating follistatin, synchronized or staggered delivery could optimize therapeutic outcomes. Initial stabilization of muscle tissue might allow subsequent treatments to be more effective.

Precise regulation of gene upregulation would be crucial to avoid adverse effects. This could be achieved using inducible promoters, which allows for the fine control of gene expression. For instance, a Tet-on/Tet-off system can turn gene expression on or off in response to the administration of tetracycline or its derivatives, thus providing precise control over the timing and level of gene expression. However, at present, such inducible systems are not clinically deployable; basal leak, limited dynamic range, added vector burden, and immunogenic regulator proteins (which may exacerbate dose-related toxicity at higher AAV exposures), together with the pharmacology and immunogenicity of small-molecule inducers and the potential for off-target or ectopic activation, remain unresolved. To avoid adverse effects in other tissues, in particular the liver, muscle-specific promoters and engineered AAV capsids to provide more highly specific targeting of skeletal muscle is now the standard for gene addition therapies for MD. However, some clinical trials such as follistatin gene transfer have used ubiquitous promoters such as CMV [55]. It might also be possible to use miR-122 to de-target dCas9 expression from the liver [159]. This strategy helps avoid potential adverse effects on other tissues, such as the heart and liver, where the upregulation of certain genes might have detrimental consequences. For instance, excessive expression of muscle-regenerative genes in the heart could lead to cardiac hypertrophy, while in the liver, it could disrupt metabolic processes.

The simultaneous upregulation of utrophin, follistatin, GALGT2, and Klotho may offer a multifaceted therapeutic approach targeting dystrophin compensation, fibrosis inhibition, and membrane stabilization, though the interplay of these pathways in vivo remains to be fully elucidated. Utrophin upregulation would compensate for varying degrees for dystrophin function, performing a crucial role in preserving muscle integrity while follistatin would inhibit fibrosis and promote muscle growth and Klotho would add muscle-protective and anti-aging benefits to stem cell function, enhancing overall muscle health. GALGT2 would theoretically stabilize the sarcolemma and prevent contraction-induced damage. This combined strategy targets multiple aspects of DMD pathology to enhance muscle growth, reduce fibrosis, and limit damage, making it a potentially universal approach for patients across different genotypes.

It would also be possible to combine follistatin and/or Klotho upregulation with the mutation-specific dystrophin targeting therapies exon skipping AOs and stop codon readthrough. The upregulated follistatin and/or Klotho would improve muscle phenotype and thereby act to enhance the efficacy and function of the restored dystrophin protein. Myostatin inhibition through the upregulation of follistatin would promote muscle growth and reduce fibrosis, exon skipping, or stop codon readthrough which can correct specific mutations in the *DMD* gene, and Klotho enhances muscle protection and function. This multi-faceted approach would ensure muscle growth and functional improvement while addressing specific genetic mutations.

CRISPRa could also be used in combination with AAV-microdystrophin gene therapy. This would only be possible if the CRISPRa machinery is delivered using a nanoparticle, as treating a patient with two AAV vectors would be difficult. Packaging either plasmid DNA, mRNA, or RNP in a nanoparticle would only give short-term expression of the dCas9 and effectors, meaning the efficacy of upregulation would be short-lived. Transient upregulation, potentially with repeat nanoparticle treatment, could be used to preserve muscle form and function through protection against muscular atrophy and fibrosis. When used prior to gene addition therapy administration, higher transduction efficiency of the AAV vector and the heightened benefit of the expressed microdystrophin protein might be achieved. The same would be true for the use of CRISPRa delivered using an AAV when combined with later AO exon skip or Ataluren readthrough. If dystrophin restoration therapy is used first the muscle would be protected from further damage and subsequent follistatin and Klotho upregulation, achieved with the appropriate delivery method, which might reverse the fibrosis seen and promote muscle growth. The order of administration would most likely be determined by the stage of the disease. Recent analysis of three publicly available independent microarray datasets and a comparison of datasets from DMD muscle and normal muscle tissues have revealed a number of differentially expressed genes that would represent potential targets for CRISPRa [160]. Identified downregulated genes (*ASB2*, *SAR1B*, *LEPREL1*) lend themselves to upregulation by CRISPRa. In another study, gene expression profiles of vastus lateralis biopsy samples from 17 patients with DMD identified several hub genes such as *C3AR1*, *TLR7, IRF8*, *FYB*, *CD33* (immune and inflammation associated genes), *TYROBP*, *PLEK*, *AIF1* (actin reorganization associated genes), *LAPTM5,* and *NT5E* (cell death and arterial calcification associated genes, respectively), as being affected as a result of the disease [161]. Within these gene hubs, 189 downregulated differentially expressed genes were noted. It appears that genes have altered expression at different stages of DMD progression. Early changes seem to be related to muscle degeneration/regeneration, while later changes are related to immune dysfunction, ECM remodeling, and myogenic dysfunction [162,163]. Much work would be needed to identify which targets would hold the highest potential for benefit, and at which stage of disease pathogenesis this benefit might be realized. It might be envisaged that with development, the future might hold the prospect of dCas9 with transcriptional activators being delivered using AAV vectors, giving long-term episomal expression. Nanoparticles might then be used to deliver sgRNAs, targeting relevant genes as appropriate at different stages of the disease.

While genome editing offers the potential for permanent correction of genetic mutations, CRISPRa presents a safer alternative due to the absence of double-strand breaks (DSBs). This reduces the risk of unintended off-target effects and genomic instability, likely leading to quicker approvals for clinical trials. CRISPRa is also non-mutation-specific, making it broadly applicable across different DMD mutations, unlike genome editing, AO exon skipping, and stop codon readthrough therapies, which are mutation-specific.

## 10. Hurdles to Be Addressed

### 10.1. Delivery of the CRISPRa Machinery

For the systemic delivery required for an effective therapy for DMD, CRISPRa systems can be packaged into viral and non-viral vectors. Viral vectors offer high delivery efficiency and the potential for long-term gene expression, crucial for therapeutic efficacy, although they have limitations when it comes to packaging capacity and have safety issues as described above for AAV vector microdystrophin trials [164,165]. These vectors can be engineered for targeted delivery and have established manufacturing processes conducive to clinical translation. In contrast, non-viral vectors like lipid nanoparticles and synthetic polymers are advancing rapidly due to their improved safety profiles and ease of manufacturing, although they may require enhancements in delivery efficiency and long-term expression [166,167].

Choosing between viral and non-viral vectors generally depends on factors such as target cell specificity, scalability, regulatory considerations, therapeutic goals, and in the case of CRISPRa, the type of Cas enzyme and system to be used [168,169]. The Cas enzyme has many different types with different gene sizes which makes some more packageable than others. The widely used *Staphylococcus. pyogenes* Cas9 (*Sp*Cas9) has a gene size of 4.1 kb, which makes it one of the largest Cas enzymes [170]. *Staphylococcus aureus* Cas9 (*Sa*Cas9) is the next popular Cas enzyme with a 3.1 kb gene size [170]. CasX found in *Deltaproteobacteria* and *Campylobacter jejuni* Cas9 (*Cj*Cas9) is the smallest identified Cas enzyme, each with 2.9 kb gene sizes [171,172]. Each of these enzymes have their own distinct protospacer adjacent motif (PAM) sequence. *Sp*Cas9, CasX, *Sa*Cas9, and *Cj*Cas9 have 5′-NGG-3′, 5′-TTCN-3′, 5′-NNGRRT-3′, and 5′-NNNNRYAC-3′ PAMs, respectively, which makes them less targetable in the same order due to their PAM complexities [170,171,172,173].

AAV vectors have a packaging capacity of 4.8–5.2 kb which makes it a perfect vector for smaller Cas cDNAs, like *Sa*Cas9, CasX, and *Cj*Cas9 but packaging larger Cas cDNA like that for *Sp*Cas9 into AAV vectors is not easy [37,174]. As a result, a novel approach for the delivery of *Sp*Cas9 using dual hybrid AAV vectors has been developed [175]. In an in vitro study, the *Sp*Cas9 gene was split in half and fused to a split intein, and each half was packaged into separate AAV vectors which were used to transduce HEK293T and Neuro-2a cells [176]. Upon delivery, these fragments are expressed, and the introns facilitate protein splicing by excising themselves and ligating the Cas9 fragments together via Homologous Directed Recombination (HDR) cell machinery. This recombination reconstitutes a functional Cas9 enzyme, enabling it to perform genome editing tasks as directed by a guide RNA. However, translationally, treating a patient with two AAV vectors would be difficult [177]. To keep to doses that would not carry risk of serious adverse events, the dose of each vector would be low and the overall expression of the dCas may not be enough to provide the upregulation needed [178,179]. To overcome these limitations, many different strategies have been explored. In response to the packaging limitation, one approach was to use more compact Cas9 orthologs such as SaCas9, CasX, or CjCas9, which are all significantly smaller than the original SpCas9 and packageable within a single AAV vector in the CRISPRa context [170,172,180]. When it comes to immunogenicity, scientists have developed engineered capsids like MyoAAV which have shown increased selectivity and tropism for skeletal muscle, while use of tissue-specific promoters and micro-RNA response elements like miR-122 allow controlled and regulated tissue-specific expression [159,181]. The more targeted efficiency and more controlled expression specific to muscle tissue is important as it allows a lower dose of AAV vector to be administered which reduces post-administration immunogenicity [182,183]. Furthermore, capsid engineering can directly reduce immunogenicity of AAV vectors. Researchers have strategically modified the surface proteins of the AAV capsid to remove or alter immunodominant epitopes that are recognized by the host’s immune system, such as neutralizing antibodies (NAbs) and cytotoxic T lymphocytes (CTLs). This immune evasion and immune-silencing of the capsid may allow the AAV treatment to overcome pre-existing immunity and allow re-administration if needed [184,185,186].

Assuming similar high dosing of 1–2 × 10^14^ vg/kg (vector genomes per kg), as used in the microdystrophin trials, would be required for the transduction of bodywide muscles, the risks of immunotoxicity and genotoxicity would be as high as seen in those trials. This would therefore be unlikely to provide any additional benefit over and above microdystrophin AAV vector, except for the fact that it would be possible to multiplex which genes might be activated together, for example, utrophin and follistatin or GALGT2 and follistatin. It should be noted that advances in engineered myotropic and liver-detargeted AAV capsids have demonstrated improved muscle transduction efficiency in preclinical models, raising the possibility that future CRISPRa approaches could achieve therapeutic benefit at lower vector doses, thereby reducing the associated immunotoxicity and genotoxicity risks.

Non-viral delivery systems have garnered significant attention in gene therapy due to their potential for overcoming challenges associated with viral vectors. Recent studies have highlighted innovative strategies in this field. For instance, recent studies have shown that serum extracellular vesicles (sEVs) enhance delivery of CRISPR-Cas9 ribonucleoprotein (RNPs) to muscle cells [187]. This approach successfully deleted mutated exons 23 and 24 in the *Dmd* gene, leading to the restoration of its expression and demonstrating efficient transfection in vivo [187]. Additionally, it has been reported that biodegradable polymeric nanoparticles are capable of encapsulating and delivering CRISPR-Cas9 RNP components to muscle with high specificity and low immunogenicity, thereby achieving precise genome editing outcomes [188]. Building on this progress, a recent study demonstrated the use of pH-sensitive hollow mesoporous silica nanoparticles, functionalized with PLZ4 ligands, to deliver the CRISPR/dCas9-SAM system for multiplex gene activation in vivo [189]. This non-viral platform enabled the efficient co-delivery of multiple plasmid components, achieving targeted gene activation and enhanced anti-tumor effects in a bladder cancer model. These findings further validate the adaptability of nanoparticle-based systems for complex CRISPR applications beyond gene editing, reinforcing their promise as scalable and safe alternatives to viral vectors. These advancements underscore the versatility of nanoparticle-based platforms in facilitating safe and effective gene therapy applications, offering promising alternatives to traditional viral vectors for CRISPRa to muscle fibers [190].

Lipid nanoparticles (LNPs) facilitate the transient expression of CRISPRa with lower immunogenicity and also allows the potential for repeated dosing unlike AAV delivery methods. Several research groups have shown that LNPs can effectively deliver Cas9 ribonucleoprotein (RNP) complex and mRNA to muscle in vivo for DMD treatment [188,190,191]. However, LNPs have low delivery efficiency and treatability relative to AAV vectors, especially when administered in large mammals. Other limitations of LNPs are accumulation in the liver and lack of specific extrahepatic targeting [192,193]. Although there has been some progress in addressing these issues, more work is required to improve payload capacity and explore more precise tissue targetability by using engineered LNPs with specific ligands or antibodies to target muscle tissue [194].

### 10.2. Experimental Model Considerations

Just as delivery strategies are important for CRISPRa systems, choosing different animal models plays a pivotal role in testing the safety, efficiency, and biological outcomes of CRISPRa therapies. In vitro platforms derived from human DMD patient pluripotent stem cells (iPSCs) differentiated into skeletal muscle are now one of the best models for the assessment of transcriptional activation and off-target effects of CRISPRa systems designed in human cells. IPSCs provide a human genetic context and have the added advantage of capturing inter-patient variability. Notably, different iPSC lines exhibit variation in myogenic differentiation potential, metabolic profile, and disease severity, even when derived from patients with similar mutations [195]. These differences can influence CRISPRa responsiveness, meaning that results from one patient-derived line may not directly translate to another. Even though IPSCs are a great platform for having the same genetic content as humans, iPSC-derived myotubes cannot replicate systemic aspects of DMD, such as inflammation, fibrosis, or long-term regeneration, and are limited in their ability to reflect delivery kinetics [196,197]. As a result, they are most effective when used for early stage target validation before moving into whole-organism models.

Rodent models such as the *mdx* mouse carrying exon 23 mutation of its *Dmd* gene are the most commonly used models for DMD studies [198]. It is also worth mentioning that there are other mouse-carrying human exons 8–34 and 45 deletions [199,200]. However, the phenotype is relatively mild and lacks many of the progressive features seen in human patients. This has driven efforts to engineer rodent lines with more severe disease phenotypes to better match human pathology. To bridge this gap, more severe models like *mdx* utrophin−/− double knockouts have been developed, which exhibit increased muscle wasting, fibrosis, and a more aggressive disease course [201]. In addition, the DMDdel52 rat model which has a deleted exon 52 of *DMD* gene is a model that resembles human DMD phenotypes more accurately when it comes to severity and fibrosis [202]. The inclusion of a humanized exon is particularly valuable for preclinical AO optimization studies. Lastly, cDMDR mutated-rats have also been generated which have exon 23 mutation and exons 3 and 16 mutations in *DMD* genes which also exhibited similar DMD-related phenotypes to humans in features like muscle fibrosis, fatty infiltration, cardiac complication, and loss of muscle force generation [203,204,205]. Together, these rodent models create a scalable and genetically diverse platform, but none can fully recapitulate the progressive and heterogeneous course of the human disease.

There are larger animal models that mimic the DMD condition better than the rodent DMD models such as the GRMD dog, DMD pig, and DMD-edited microminipigs. These models are known to show the most faithful mimic of the human DMD phenotype. GRMD dog model has a mutation in intron 6 which results in skipping of exon 7 during mRNA processing and DMD-edited microminipigs have an exon 23 mutation while the DMD pig model has exon 52 deletion [206,207,208]. GRMD dogs show notable phenotype variation, with some animals exhibiting severe, early onset weakness and rapid progression, while others present with milder symptoms and slower disease course. Such variability is clinically important, as it mirrors the patient-to-patient differences seen in human DMD, which can affect both disease prognosis and therapeutic response. This variability is influenced by genetic modifiers such as LTBP4 and Jagged1, as well as environmental factors, and it reflects the heterogeneity seen in human patients [209]. GRMD dog, DMD pig, and DMD-edited microminipigs show progressive weakness, respiratory dysfunction, and cardiomyopathy, and they represent important models for evaluating the systemic delivery of gene therapies and immune response towards the treatment in a more clinically representative setting [206,207,208]. However, these larger models come with limitations such as expensive maintenance, longer experimental timelines, and ethical considerations. Despite these drawbacks, their size, physiology, and disease course make them indispensable for bridging the gap between preclinical work and clinical application.

Even though these models have shown to be critical to gene therapy research for DMD and have a wide range of mutations to mimic the disease, it is important to recognize that all these models have limitations when it comes to CRISPRa preclinical studies. Most notably, there are differences in genome sequences, specifically in the gene regulatory regions of each animal model relative to humans, which means that sgRNAs designed for one species are not necessarily transferable to another. This can lead to significant discrepancies between species in CRISPRa target engagement and gene activation efficiency. For example, an sgRNA that activates a gene in mouse may completely miss its target in human cells due to subtle differences in the promoter sequence or enhancer location. This necessitates the use of parallel sgRNA design and validation pipelines tailored to each species which is good for proof of concept but does not directly relate to the specific sgRNA designed for the human gene. Furthermore, the only model that offers human relevance is patient iPSC-derived cells, but they lack the systemic interactions of a living organism. Conversely, animal models allow for the evaluation of systemic effects but may not fully represent the human molecular context. Therefore, relying on a single model carries the risk of drawing incomplete or misleading conclusions. The most reliable preclinical strategy is therefore to integrate multiple models that complement each other’s strengths and compensate for their individual weaknesses.

To address this, a staged validation pipeline is needed. This pipeline, beginning with in vitro human cell models, progressing through small and large animal models, and ultimately integrating genomic and transcriptomic analysis is essential for robust translation. Careful integration of delivery method optimization and model system selection will be key to moving CRISPRa-based DMD therapies from proof of concept to clinical applications. This also shows the need for animal models carrying DMD human mutations with target genes that have human promoter regions to make CRISPRa preclinical studies more translatable to humans and more than just a proof of concept. Such models would represent a significant step forward in closing the gap between experimental feasibility and therapeutic translation.

### 10.3. Immunity to Cas9

As Cas’s are bacterial proteins, pre-existing immunity in patients is likely a result of previous infections. This can include both antibody responses and T cell responses, with Cas9-specific cytotoxic T-cells able to remove transduced cells. A study that screened human serum found that nearly 80% of donors have anti-SaCas9 antibodies, while antibodies to SpCas9 were present in nearly 60% of donors [210]. T-cell reactivity against SaCas9 has also been detected in healthy individuals, which means both antibody and T-cell immunity could limit how much editing or upregulation is achieved and how long they last [211]. This might be overcome through the use of immune suppression using pharmacological intervention or by use of Tregs. Other ways to manage this include short-term delivery of Cas9 as mRNA or ribonucleoprotein to limit how long it is present, chemical changes to guide RNAs to reduce innate immune sensing, and engineering Cas9 variants with fewer immunogenic epitopes [212]. Both SaCas9 and SpCas9 are now in clinical trial with no reported issues in relation to immune reaction to the Cas protein, implying that the anti-Cas9 immunity is manageable. Screening patients for pre-existing cellular immunity, optimizing delivery methods to limit inflammatory responses, and targeting immune-privileged tissues where possible could help maintain safety and efficacy in future applications.

### 10.4. Limitations of CRISPRa

Despite its remarkable versatility and promising potential, CRISPRa technology still faces several limitations. One major challenge is the incomplete understanding of how target gene expression levels are controlled. Specifically, even when a sgRNA successfully directs the CRISPRa machinery to the intended genomic locus, significant upregulation of the target gene does not always occur. Additionally, the degree of gene activation is largely uncontrollable by the user, raising concerns about potentially harmful overexpression. Conversely, in many cases, the level of induction remains modest due to the inherently variable efficiency of CRISPRa systems. Another practical limitation is the challenge of delivering the relatively large CRISPRa constructs into cells, restricting its application in hard-to-transfect cell types. Unlike CRISPR-mediated gene knockout, which can be achieved with transient Cas/sgRNA expression, CRISPRa requires continuous expression of dCas and sgRNA to sustain target gene activation. As noted earlier, a limitation of CRISPRa is the large size of its constructs and the need for additional activating elements, which challenges efficient delivery. The conventional SpCas9, for example, is the most studied subtype bypassing the packaging capacity of AAV vectors. Nonetheless, recently a dCasMINI-VPR system has been used to target utrophin in *mdx* mice with multiplexed sgRNA for the increased upregulation of expression [157]. For human cells, integrating multiple components remains advantageous. To this end, a piggyBac-based CRISPRa system (CAG-CRISPRa-sel) was developed, containing all necessary components for efficient activation. Driven by a strong CAG promoter and coupled with puromycin selection, this system induced the robust expression of target genes across ten commonly used human cell lines [213]. Within a clinical framework, piggyBac-based CRISPRa constructs are primarily in vitro benchmarking platforms rather than candidates for therapeutic delivery; their reliance on genomic integration, antibiotic selection, and constitutive CAG-driven expression renders them unsuitable for systemic applications in DMD. Their value is in comparing activator architectures and multiplex guide designs prior to translation into non-integrating modalities (e.g., compact AAV vectors or transient mRNA/LNP) that align with dose and safety constraints.

## 11. Conclusions

In summary, CRISPRa may offer a flexible platform that may complement existing gene therapies for DMD, particularly when applied in a context-specific and temporally regulated manner. Integrating CRISPRa with strategies such as exon skipping or micro-dystrophin gene addition may have the potential to address both the primary and secondary pathological mechanisms of the disease. By improving the overall muscle environment through reduced fibrosis and enhanced regeneration, the therapeutic impact of dystrophin-restoring approaches could be amplified, potentially allowing for reduced vector doses and improved patient outcomes. Moving forward, it is essential to evaluate the combinatorial effects of multi-target upregulation and to establish precise control over gene expression levels, ensuring that therapeutic benefits are achieved without inducing off-target consequences. CRISPRa has the potential to be a useful tool in the treatment of DMD but there is much to optimize and many major hurdles to be addressed before any benefit might be realized.

## Data Availability

No new data were created or analyzed in this study.

## Abbreviations

The following abbreviations are used in this manuscript:

FDA	Food and Drug Administration
EMA	European Medicines Agency
ASO	Antisense Oligonucleotide
nNOS	Neuronal Nitric Oxide Synthase
ERK	Extracellular Signal-regulated Kinase
UTRN	Utrophin
GALGT2 (B4GALNT2)	Beta-1,4-N-acetylgalactosaminyltransferase 2
Cas9	CRISPR nucleases
dCas9	Dead Cas9
VP64, VPR, p65, Rta	Transcriptional Activator Domains
miRNA	MicroRNA
sgRNA	Single Stranded Guide RNA
DMD	Duchenne Muscular Dystrophy
BMD	Becker Muscular Dystrophy
AAV	Adeno-associated Virus
CRISPRa	CRISPR Activation
NSAA	North Star Ambulatory Assessment
6MWT	6 min Walk Test
SAE	Serious Adverse Event
CMV	Cytomegalovirus (promoter)
MCK	Muscle Creatine Kinase (promoter)
α-DG	Alpha-dystroglycan
cDNA	Complementary DNA
HDR	Homology-directed Repair
PAM	Protospacer Adjacent Motif
FS344/FS315	Follistatin Isoforms

## References

[1] Lovering R.M., Porter N.C., Bloch R.J. (2005). The muscular dystrophies: From genes to therapies. Phys. Ther..

[2] Draušnik Ž., Cerovečki I., Štefančić V., Mihel S., Stevanović R., Barišić N., Matković H., Melša M., Mirić M., Pjevač N. (2019). The prevalence of muscular dystrophy and spinal muscular atrophy in Croatia: Data from national and non-governmental organization registries. Croat. Med. J..

[3] Nguyen C.-T.E., Campbell C. (2016). Myotonic dystrophy type 1. Can. Med. Assoc. J..

[4] Crisafulli S., Sultana J., Fontana A., Salvo F., Messina S., Trifirò G. (2020). Global epidemiology of Duchenne muscular dystrophy: An updated systematic review and meta-analysis. Orphanet J. Rare Dis..

[5] Theadom A., Rodrigues M., Roxburgh R., Balalla S., Higgins C., Bhattacharjee R., Jones K., Krishnamurthi R., Feigin V. (2014). Prevalence of Muscular Dystrophies: A Systematic Literature Review. Neuroepidemiology.

[6] Deenen J.C.W., Arnts H., van der Maarel S.M., Padberg G.W., Verschuuren J.J.G.M., Bakker E., Weinreich S.S., Verbeek A.L.M., van Engelen B.G.M. (2014). Population-based incidence and prevalence of facioscapulohumeral dystrophy. Neurology.

[7] Giardina E., Camaño P., Burton-Jones S., Ravenscroft G., Henning F., Magdinier F., van der Stoep N., van der Vliet P.J., Bernard R., Tomaselli P.J. (2024). Best practice guidelines on genetic diagnostics of facioscapulohumeral muscular dystrophy: Update of the 2012 guidelines. Clin. Genet..

[8] Nowak K.J., Davies K.E. (2004). Duchenne muscular dystrophy and dystrophin: Pathogenesis and opportunities for treatment. EMBO Rep..

[9] Gao Q.Q., McNally E.M. (2015). The Dystrophin Complex: Structure, Function, and Implications for Therapy. Compr. Physiol..

[10] Petrof B.J., Shrager J.B., Stedman H.H., Kelly A.M., Sweeney H.L. (1993). Dystrophin protects the sarcolemma from stresses developed during muscle contraction. Proc. Natl. Acad. Sci. USA.

[11] Mareedu S., Million E.D., Duan D., Babu G.J. (2021). Abnormal Calcium Handling in Duchenne Muscular Dystrophy: Mechanisms and Potential Therapies. Front. Physiol..

[12] Boldrin L., Zammit P.S., Morgan J.E. (2015). Satellite cells from dystrophic muscle retain regenerative capacity. Stem Cell Res..

[13] Sahani R., Wallace C.H., Jones B.K., Blemker S.S. (2022). Diaphragm muscle fibrosis involves changes in collagen organization with mechanical implications in Duchenne muscular dystrophy. J. Appl. Physiol..

[14] Kharraz Y., Guerra J., Pessina P., Serrano A.L., Muñoz-Cánoves P. (2014). Understanding the process of fibrosis in Duchenne muscular dystrophy. BioMed Res. Int..

[15] Guiraud S., Roblin D., Kay D.E. (2018). The potential of utrophin modulators for the treatment of Duchenne muscular dystrophy. Expert Opin. Orphan Drugs.

[16] Yucel N., Chang A.C., Day J.W., Rosenthal N., Blau H.M. (2018). Humanizing the mdx mouse model of DMD: The long and the short of it. npj Regen. Med..

[17] Yao S., Chen Z., Yu Y., Zhang N., Jiang H., Zhang G., Zhang Z., Zhang B. (2021). Current Pharmacological Strategies for Duchenne Muscular Dystrophy. Front. Cell Dev. Biol..

[18] Matthews E., Brassington R., Kuntzer T., Jichi F., Manzur A.Y. (2016). Corticosteroids for the treatment of Duchenne muscular dystrophy. Cochrane Database Syst. Rev..

[19] Hammer S., Toussaint M., Vollsæter M., Nesbjørg Tvedt M., Drange Røksund O., Reychler G., Lund H., Andersen T. (2022). Exercise Training in Duchenne Muscular Dystrophy: A Systematic Review and Meta-Analysis. J. Rehabil. Med..

[20] Buzello W., Huttarsch H. (1988). Muscle Relaxation in Patients with Duchenne’s Muscular Dystrophy: Use of Vecuronium in Two Patients. Br. J. Anaesth..

[21] Forst J., Forst R. (2012). Surgical treatment of Duchenne muscular dystrophy patients in Germany: The present situation. Acta Myol..

[22] Muntoni F., Desguerre I., Guglieri M., Osorio A.N., Kirschner J., Tulinius M., Buccella F., Elfring G., Werner C., Schilling T. (2019). Ataluren use in patients with nonsense mutation Duchenne muscular dystrophy: Patient demographics and characteristics from the STRIDE Registry. J. Comp. Eff. Res..

[23] Ng M.Y., Li H., Ghelfi M.D., Goldman Y.E., Cooperman B.S. (2021). Ataluren and aminoglycosides stimulate read-through of nonsense codons by orthogonal mechanisms. Proc. Natl. Acad. Sci. USA.

[24] Finkel R.S., Flanigan K.M., Wong B., Bönnemann C., Sampson J., Sweeney H.L., Reha A., Northcutt V.J., Elfring G., Barth J. (2013). Phase 2a Study of Ataluren-Mediated Dystrophin Production in Patients with Nonsense Mutation Duchenne Muscular Dystrophy. PLoS ONE.

[25] Wells D.J. (2019). What is the level of dystrophin expression required for effective therapy of Duchenne muscular dystrophy?. J. Muscle Res. Cell Motil..

[26] Politano L. (2021). Read-through approach for stop mutations in Duchenne muscular dystrophy. An update. Acta Myol..

[27] European Medicines Agency (2024). Translarna: EMA Re-Confirms Non-Renewal of Authorisation of Duchenne Muscular Dystrophy Medicine. https://www.ema.europa.eu/en/news/translarna-ema-re-confirms-non-renewal-authorisation-duchenne-muscular-dystrophy-medicine.

[28] Nakamura A. (2017). Moving towards successful exon-skipping therapy for Duchenne muscular dystrophy. J. Hum. Genet..

[29] Aartsma-Rus A., van Ommen G.J. (2007). Antisense-mediated exon skipping: A versatile tool with therapeutic and research applications. RNA.

[30] Aartsma-Rus A., De Waele L., Houwen-Opstal S., Kirschner J., Krom Y.D., Mercuri E., Niks E.H., Straub V., van Duyvenvoorde H.A., Vroom E. (2023). The Dilemma of Choice for Duchenne Patients Eligible for Exon 51 Skipping the European Experience. J. Neuromuscul. Dis..

[31] Tyagi R., Kumar S., Dalal A., Mohammed F., Mohanty M., Kaur P., Anand A. (2019). Repurposing Pathogenic Variants of *DMD* Gene and its Isoforms for DMD Exon Skipping Intervention. Curr. Genom..

[32] Scaglioni D., Catapano F., Ellis M., Torelli S., Chambers D., Feng L., Beck M., Sewry C., Monforte M., Harriman S. (2021). The administration of antisense oligonucleotide golodirsen reduces pathological regeneration in patients with Duchenne muscular dystrophy. Acta Neuropathol. Commun..

[33] Eser G., Topaloğlu H. (2022). Current Outline of Exon Skipping Trials in Duchenne Muscular Dystrophy. Genes.

[34] Hoffman E.P., McNally E.M. (2014). Exon-skipping therapy: A roadblock, detour, or bump in the road?. Sci. Transl. Med..

[35] Mitelman O., Abdel-Hamid H.Z., Byrne B.J., Connolly A.M., Heydemann P., Proud C., Shieh P.B., Wagner K.R., Dugar A., Santra S. (2022). A Combined Prospective and Retrospective Comparison of Long-Term Functional Outcomes Suggests Delayed Loss of Ambulation and Pulmonary Decline with Long-Term Eteplirsen Treatment. J. Neuromuscul. Dis..

[36] Mendell J.R., Khan N., Sha N., Eliopoulos H., McDonald C.M., Goemans N., Mercuri E., Lowes L.P., Alfano L.N. (2021). Comparison of Long-term Ambulatory Function in Patients with Duchenne Muscular Dystrophy Treated with Eteplirsen and Matched Natural History Controls. J. Neuromuscul. Dis..

[37] Naso M.F., Tomkowicz B., Perry W.L., Strohl W.R. (2017). Adeno-Associated Virus (AAV) as a Vector for Gene Therapy. BioDrugs.

[38] Mullard A. (2023). FDA approves first gene therapy for Duchenne muscular dystrophy, despite internal objections. Nat. Rev. Drug Discov..

[39] Duan D. (2018). Systemic AAV Micro-dystrophin Gene Therapy for Duchenne Muscular Dystrophy. Mol. Ther..

[40] Cernisova V., Lu-Nguyen N., Trundle J., Herath S., Malerba A., Popplewell L. (2023). Microdystrophin Gene Addition Significantly Improves Muscle Functionality and Diaphragm Muscle Histopathology in a Fibrotic Mouse Model of Duchenne Muscular Dystrophy. Int. J. Mol. Sci..

[41] Wilton-Clark H., Yokota T. (2022). Antisense and Gene Therapy Options for Duchenne Muscular Dystrophy Arising from Mutations in the N-Terminal Hotspot. Genes.

[42] Hart C.C., Lee Y.i., Xie J., Gao G., Lin B.L., Hammers D.W., Sweeney H.L. (2024). Potential limitations of microdystrophin gene therapy for Duchenne muscular dystrophy. JCI Insight.

[43] Łoboda A., Dulak J. (2020). Muscle and cardiac therapeutic strategies for Duchenne muscular dystrophy: Past, present, and future. Pharmacol. Rep..

[44] Silver E., Argiro A., Hong K., Adler E. (2024). Gene therapy vector-related myocarditis. Int. J. Cardiol..

[45] Howard Z.M., Dorn L.E., Lowe J., Gertzen M.D., Ciccone P., Rastogi N., Odom G.L., Accornero F., Chamberlain J.S., Rafael-Fortney J.A. (2021). Micro-dystrophin gene therapy prevents heart failure in an improved Duchenne muscular dystrophy cardiomyopathy mouse model. JCI Insight.

[46] Forand A., Moog S., Mougenot N., Lemaitre M., Sevoz-Couche C., Guesmia Z., Virtanen L., Giordani L., Muchir A., Pietri-Rouxel F. (2025). Long-Term Dystrophin Replacement Therapy in Duchenne Muscular Dystrophy Causes Cardiac Inflammation. JACC Basic Transl. Sci..

[47] Mendell J.R., Proud C., Zaidman C.M., Mason S., Darton E., Wang S., Wandel C., Murphy A.P., Mercuri E., Muntoni F. (2024). Practical Considerations for Delandistrogene Moxeparvovec Gene Therapy in Patients with Duchenne Muscular Dystrophy. Pediatr. Neurol..

[48] Hoy S.M. (2023). Delandistrogene Moxeparvovec: First Approval. Drugs.

[49] Muntoni F., Mercuri E., Schmidt U.S., Komaki H., Richardson J., Singh T., Guridi M., Mason S., Murphy A., Yu L. (2023). EMBARK, a Phase 3 Trial Evaluating Safety and Efficacy of Delandistrogene Moxeparvovec (SRP-9001) in Duchenne Muscular Dystrophy (DMD): Study Design and Baseline Characteristics (P5-8.012). Neurology.

[50] Sarepta Therapeutics I. Sarepta Therapeutics Announces Voluntary Pause of ELEVIDYS Shipments in the U.S. https://investorrelations.sarepta.com/news-releases/news-release-details/sarepta-therapeutics-announces-voluntary-pause-elevidys.

[51] Dastgir J., Falabella P., Qiao C., Kim S., Buss N., Fiscella M., Pakola S., Danos O. (2022). P.130 RGX-202: An investigational AAV8 gene therapy coding for a novel microdystrophin as a treatment for Duchenne muscular dystrophy. Neuromuscul. Disord..

[52] Roberts T.C., Wood M.J.A., Davies K.E. (2023). Therapeutic approaches for Duchenne muscular dystrophy. Nat. Rev. Drug Discov..

[53] Laugel V., De Lucia S., Davion J., Daniele N., Cao F., Sanz M., Buscara L., Blaie S., Thibaut L., Sagot M. (2024). 410P GNT0004, Genethon’s AAV8 vector-delivered microdystrophin gene therapy of Duchenne muscular dystrophy, first data of the phase I/II part of the GNT-016-MDYF all-in-one clinical trial in ambulant boys. Neuromuscul. Disord..

[54] Flanigan K.M., Vetter T.A., Simmons T.R., Iammarino M., Frair E.C., Rinaldi F., Chicoine L.G., Harris J., Cheatham J.P., Cheatham S.L. (2022). A first-in-human phase I/IIa gene transfer clinical trial for Duchenne muscular dystrophy using rAAVrh74.MCK.*GALGT2*. Mol. Ther. Methods Clin. Dev..

[55] Mendell J.R., Sahenk Z., Malik V., Gomez A.M., Flanigan K.M., Lowes L.P., Alfano L.N., Berry K., Meadows E., Lewis S. (2015). A Phase 1/2a Follistatin Gene Therapy Trial for Becker Muscular Dystrophy. Mol. Ther..

[56] Crudele J.M., Chamberlain J.S. (2019). AAV-based gene therapies for the muscular dystrophies. Hum. Mol. Genet..

[57] Rajaganapathy S., McCourt J.L., Ghosal S., Lindsay A., McCourt P.M., Lowe D.A., Ervasti J.M., Salapaka M.V. (2019). Distinct mechanical properties in homologous spectrin-like repeats of utrophin. Sci. Rep..

[58] Soblechero-Martín P., López-Martínez A., de la Puente-Ovejero L., Vallejo-Illarramendi A., Arechavala-Gomeza V. (2021). Utrophin modulator drugs as potential therapies for Duchenne and Becker muscular dystrophies. Neuropathol. Appl. Neurobiol..

[59] Guiraud S., Davies K.E. (2017). Pharmacological advances for treatment in Duchenne muscular dystrophy. Curr. Opin. Pharmacol..

[60] Guiraud S., Chen H., Burns D.T., Davies K.E. (2015). Advances in genetic therapeutic strategies for Duchenne muscular dystrophy. Exp. Physiol..

[61] Clerk A., Morris G.E., Dubowitz V., Davies K.E., Sewry C.A. (1993). Dystrophin-related protein, utrophin, in normal and dystrophic human fetal skeletal muscle. Histochem. J..

[62] Guiraud S., Davies K. (2023). Utrophin correlates with disease severity in Duchenne muscular dystrophy. Med.

[63] Gorokhova S., Schessl J., Zou Y., Yang M.L., Heydemann P.T., Sufit R.L., Meilleur K., Donkervoort S., Medne L., Finkel R.S. (2023). Unusually severe muscular dystrophy upon in-frame deletion of the dystrophin rod domain and lack of compensation by membrane-localized utrophin. Med.

[64] Chatzopoulou M., Conole D., Emer E., Rowley J.A., Willis N.J., Squire S.E., Gill B., Brough S., Wilson F.X., Wynne G.M. (2022). Structure-activity relationships of 2-pyrimidinecarbohydrazides as utrophin modulators for the potential treatment of Duchenne muscular dystrophy. Bioorganic Med. Chem..

[65] Tinsley J.M., Fairclough R.J., Storer R., Wilkes F.J., Potter A.C., Squire S.E., Powell D.S., Cozzoli A., Capogrosso R.F., Lambert A. (2011). Daily treatment with SMTC1100, a novel small molecule utrophin upregulator, dramatically reduces the dystrophic symptoms in the mdx mouse. PLoS ONE.

[66] Markati T., De Waele L., Schara-Schmidt U., Servais L. (2021). Lessons Learned from Discontinued Clinical Developments in Duchenne Muscular Dystrophy. Front. Pharmacol..

[67] Vuorinen A., Wilkinson I.V.L., Chatzopoulou M., Edwards B., Squire S.E., Fairclough R.J., Bazan N.A., Milner J.A., Conole D., Donald J.R. (2021). Discovery and mechanism of action studies of 4,6-diphenylpyrimidine-2-carbohydrazides as utrophin modulators for the treatment of Duchenne muscular dystrophy. Eur. J. Med. Chem..

[68] Babbs A., Berg A., Chatzopoulou M., Davies K.E., Davies S.G., Edwards B., Elsey D.J., Emer E., Figuccia A.L.A., Fletcher A.M. (2020). Synthesis of SMT022357 enantiomers and in vivo evaluation in a Duchenne muscular dystrophy mouse model. Tetrahedron.

[69] Song M.H., Yoo J., Oh J.G., Kook H., Park W.J., Jeong D. (2022). Matricellular Protein CCN5 Gene Transfer Ameliorates Cardiac and Skeletal Dysfunction in mdx/utrn (±) Haploinsufficient Mice by Reducing Fibrosis and Upregulating Utrophin Expression. Front. Cardiovasc. Med..

[70] Ghosh S., Arshi M.U., Ghosh S., Jash M., Sen S., Mamchaoui K., Bhattacharyya S., Rana N.K., Ghosh S. (2024). Discovery of Quinazoline and Quinoline-Based Small Molecules as Utrophin Upregulators via AhR Antagonism for the Treatment of Duchenne Muscular Dystrophy. J. Med. Chem..

[71] Mahmoudzadeh Laki R., Pourbasheer E. (2024). 3D-QSAR Modeling on 2-Pyrimidine Carbohydrazides as Utrophin Modulators for the Treatment of Duchenne Muscular Dystrophy by Combining CoMFA, CoMSIA, and Molecular Docking Studies. ACS Omega.

[72] Babbs A., Berg A., Chatzopoulou M., Davies K.E., Davies S.G., Edwards B., Elsey D.J., Emer E., Guiraud S., Harriman S. (2020). 2-Arylbenzo[d]oxazole Phosphinate Esters as Second-Generation Modulators of Utrophin for the Treatment of Duchenne Muscular Dystrophy. J. Med. Chem..

[73] Lu Y., Tian C., Danialou G., Gilbert R., Petrof B.J., Karpati G., Nalbantoglu J. (2008). Targeting Artificial Transcription Factors to the Utrophin A Promoter: Effects on Dystrophic Pathology and Muscle Function *. J. Biol. Chem..

[74] Strimpakos G., Corbi N., Pisani C., Di Certo M.G., Onori A., Luvisetto S., Severini C., Gabanella F., Monaco L., Mattei E. (2014). Novel Adeno-Associated Viral Vector Delivering the Utrophin Gene Regulator Jazz Counteracts Dystrophic Pathology in mdx Mice. J. Cell. Physiol..

[75] Pisani C., Strimpakos G., Gabanella F., Di Certo M.G., Onori A., Severini C., Luvisetto S., Farioli-Vecchioli S., Carrozzo I., Esposito A. (2018). Utrophin up-regulation by artificial transcription factors induces muscle rescue and impacts the neuromuscular junction in mdx mice. Biochim. Biophys. Acta BBA-Mol. Basis Dis..

[76] Sengupta K., Mishra M.K., Loro E., Spencer M.J., Pyle A.D., Khurana T.S. (2020). Genome Editing-Mediated Utrophin Upregulation in Duchenne Muscular Dystrophy Stem Cells. Mol. Ther.-Nucleic Acids.

[77] Bulaklak K., Xiao B., Qiao C., Li J., Patel T., Jin Q., Li J., Xiao X. (2018). MicroRNA-206 Downregulation Improves Therapeutic Gene Expression and Motor Function in *mdx* Mice. Mol. Ther. Nucleic Acids.

[78] Sengupta K., Loro E., Khurana T.S. (2020). PMO-based let-7c site blocking oligonucleotide (SBO) mediated utrophin upregulation in mdx mice, a therapeutic approach for Duchenne muscular dystrophy (DMD). Sci. Rep..

[79] Duan D. (2019). Micro-utrophin Therapy for Duchenne Muscular Dystrophy. Mol. Ther..

[80] Starikova A.V., Skopenkova V.V., Polikarpova A.V., Reshetov D.A., Vassilieva S.G., Velyaev O.A., Shmidt A.A., Savchenko I.M., Soldatov V.O., Egorova T.V. (2022). Therapeutic potential of highly functional codon-optimized microutrophin for muscle-specific expression. Sci. Rep..

[81] Kennedy T.L., Guiraud S., Edwards B., Squire S., Moir L., Babbs A., Odom G., Golebiowski D., Schneider J., Chamberlain J.S. (2018). Micro-utrophin Improves Cardiac and Skeletal Muscle Function of Severely Affected D2/*mdx* Mice. Mol. Ther. Methods Clin. Dev..

[82] Song Y., Morales L., Malik A.S., Mead A.F., Greer C.D., Mitchell M.A., Petrov M.T., Su L.T., Choi M.E., Rosenblum S.T. (2019). Non-immunogenic utrophin gene therapy for the treatment of muscular dystrophy animal models. Nat. Med..

[83] Banks G.B., Chamberlain J.S., Odom G.L. (2020). Microutrophin expression in dystrophic mice displays myofiber type differences in therapeutic effects. PLoS Genet..

[84] Odom G.L., Gregorevic P., Allen J.M., Finn E., Chamberlain J.S. (2008). Microutrophin Delivery Through rAAV6 Increases Lifespan and Improves Muscle Function in Dystrophic Dystrophin/Utrophin-deficient Mice. Mol. Ther..

[85] Florczyk-Soluch U., Polak K., Dulak J. (2021). The multifaceted view of heart problem in Duchenne muscular dystrophy. Cell. Mol. Life Sci..

[86] Flori L., Testai L., Calderone V. (2021). The “irisin system”: From biological roles to pharmacological and nutraceutical perspectives. Life Sci..

[87] Al-Zaidy S.A., Sahenk Z., Rodino-Klapac L.R., Kaspar B., Mendell J.R. (2015). Follistatin Gene Therapy Improves Ambulation in Becker Muscular Dystrophy. J. Neuromuscul. Dis..

[88] Zhu J., Li Y., Lu A., Gharaibeh B., Ma J., Kobayashi T., Quintero A.J., Huard J. (2011). Follistatin Improves Skeletal Muscle Healing after Injury and Disease through an Interaction with Muscle Regeneration, Angiogenesis, and Fibrosis. Am. J. Pathol..

[89] Nguyen H.Q., Iskenderian A., Ehmann D., Jasper P., Zhang Z., Rong H., Welty D., Narayanan R. (2020). Leveraging Quantitative Systems Pharmacology Approach into Development of Human Recombinant Follistatin Fusion Protein for Duchenne Muscular Dystrophy. CPT: Pharmacomet. Syst. Pharmacol..

[90] Pearsall R.S., Davies M.V., Cannell M., Li J., Widrick J., Mulivor A.W., Wallner S., Troy M.E., Spaits M., Liharska K. (2019). Follistatin-based ligand trap ACE-083 induces localized hypertrophy of skeletal muscle with functional improvement in models of neuromuscular disease. Sci. Rep..

[91] Shen C., Iskenderian A., Lundberg D., He T., Palmieri K., Crooker R., Deng Q., Traylor M., Gu S., Rong H. (2018). Protein Engineering on Human Recombinant Follistatin: Enhancing Pharmacokinetic Characteristics for Therapeutic Application. J. Pharmacol. Exp. Ther..

[92] Lodberg A. (2021). Principles of the activin receptor signaling pathway and its inhibition. Cytokine Growth Factor Rev..

[93] Chicoine L.G., Rodino-Klapac L.R., Shao G., Xu R., Bremer W.G., Camboni M., Golden B., Montgomery C.L., Shontz K., Heller K.N. (2014). Vascular Delivery of rAAVrh74.MCK.GALGT2 to the Gastrocnemius Muscle of the Rhesus Macaque Stimulates the Expression of Dystrophin and Laminin α2 Surrogates. Mol. Ther..

[94] Hoyte K., Kang C., Martin P.T. (2002). Definition of pre- and postsynaptic forms of the CT carbohydrate antigen at the neuromuscular junction: Ubiquitous expression of the CT antigens and the CT GalNAc transferase in mouse tissues. Mol. Brain Res..

[95] Nguyen H.H., Jayasinha V., Xia B., Hoyte K., Martin P.T. (2002). Overexpression of the cytotoxic T cell GalNAc transferase in skeletal muscle inhibits muscular dystrophy in mdx mice. Proc. Natl. Acad. Sci. USA.

[96] Ohlendieck K., Ervasti J.M., Matsumura K., Kahl S.D., Leveille C.J., Campbell K.P. (1991). Dystrophin-related protein is localized to neuromuscular junctions of adult skeletal muscle. Neuron.

[97] Yoon J.H., Chandrasekharan K., Xu R., Glass M., Singhal N., Martin P.T. (2009). The synaptic CT carbohydrate modulates binding and expression of extracellular matrix proteins in skeletal muscle: Partial dependence on utrophin. Mol. Cell. Neurosci..

[98] Xu R., Jia Y., Zygmunt D.A., Martin P.T. (2019). rAAVrh74.MCK.*GALGT2* Protects against Loss of Hemodynamic Function in the Aging *mdx* Mouse Heart. Mol. Ther..

[99] Xu R., Singhal N., Serinagaoglu Y., Chandrasekharan K., Joshi M., Bauer J.A., Janssen P.M.L., Martin P.T. (2015). Deletion of *Galgt2* (*B4Galnt2*) Reduces Muscle Growth in Response to Acute Injury and Increases Muscle Inflammation and Pathology in Dystrophin-Deficient Mice. Am. J. Pathol..

[100] Martin P.T., Xu R., Rodino-Klapac L.R., Oglesbay E., Camboni M., Montgomery C.L., Shontz K., Chicoine L.G., Clark K.R., Sahenk Z. (2009). Overexpression of Galgt2 in skeletal muscle prevents injury resulting from eccentric contractions in both mdx and wild-type mice. Am. J. Physiol.-Cell Physiol..

[101] Xu R., Camboni M., Martin P.T. (2007). Postnatal overexpression of the CT GalNAc transferase inhibits muscular dystrophy in mdx mice without altering muscle growth or neuromuscular development: Evidence for a utrophin-independent mechanism. Neuromuscul. Disord..

[102] Martin P.T., Zygmunt D.A., Ashbrook A., Hamilton S., Packer D., Birch S.M., Bettis A.K., Balog-Alvarez C.J., Guo L.-J., Nghiem P.P. (2021). Short-term treatment of golden retriever muscular dystrophy (GRMD) dogs with rAAVrh74.MHCK7.GALGT2 induces muscle glycosylation and utrophin expression but has no significant effect on muscle strength. PLoS ONE.

[103] Zygmunt D.A., Xu R., Jia Y., Ashbrook A., Menke C., Shao G., Yoon J.H., Hamilton S., Pisharath H., Bolon B. (2019). rAAVrh74.MCK.*GALGT2* Demonstrates Safety and Widespread Muscle Glycosylation after Intravenous Delivery in C57BL/6J Mice. Mol. Ther. Methods Clin. Dev..

[104] Beyersdorf J.P., Bawage S., Iglesias N., Peck H.E., Hobbs R.A., Wroe J.A., Zurla C., Gersbach C.A., Santangelo P.J. (2022). Robust, Durable Gene Activation In Vivo via mRNA-Encoded Activators. ACS Nano.

[105] Kim J.H., Hwang K.H., Park K.S., Kong I.D., Cha S.K. (2015). Biological Role of Anti-aging Protein Klotho. J. Lifestyle Med..

[106] Kuro-o M. (2011). Klotho and the aging process. Korean J. Intern. Med..

[107] Mota J., Lima A.M.M., Gomes J.I.S., Souza de Andrade M., Brito H.O., Silva M.M.A.L., Faustino-Rocha A.I., Oliveira P.A., Lopes F.F., Gil da Costa R.M. (2023). Klotho in Cancer: Potential Diagnostic and Prognostic Applications. Diagnostics.

[108] Buchanan S., Combet E., Stenvinkel P., Shiels P.G. (2020). Klotho, Aging, and the Failing Kidney. Front. Endocrinol..

[109] Wehling-Henricks M., Li Z., Lindsey C., Wang Y., Welc S.S., Ramos J.N., Khanlou N., Kuro-o M., Tidball J.G. (2016). Klotho gene silencing promotes pathology in the mdx mouse model of Duchenne muscular dystrophy. Hum. Mol. Genet..

[110] Phelps M., Yablonka-Reuveni Z. (2021). Female Outperformance in Voluntary Running Persists in Dystrophin-Null and Klotho-Overexpressing Mice. J. Neuromuscul. Dis..

[111] Javier A.J.S., Kennedy F.M., Yi X., Wehling-Henricks M., Tidball J.G., White K.E., Witczak C.A., Kuro-o M., Welc S.S. (2025). Klotho Is Cardioprotective in the *mdx* Mouse Model of Duchenne Muscular Dystrophy. Am. J. Pathol..

[112] Feldman A. (2025). InnovationRx: Sarepta Blinks in Showdown with FDA. Forbes.

[113] Duan D. (2023). Lethal immunotoxicity in high-dose systemic AAV therapy. Mol. Ther..

[114] Liu J., Koay T.W., Maiakovska O., Zayas M., Grimm D. (2023). Progress in Bioengineering of Myotropic Adeno-Associated Viral Gene Therapy Vectors. Hum. Gene Ther..

[115] Chan Y.K., Wang S.K., Chu C.J., Copland D.A., Letizia A.J., Costa Verdera H., Chiang J.J., Sethi M., Wang M.K., Neidermyer W.J. (2021). Engineering adeno-associated viral vectors to evade innate immune and inflammatory responses. Sci. Transl. Med..

[116] Muhuri M., Zhan W., Maeda Y., Li J., Lotun A., Chen J., Sylvia K., Dasgupta I., Arjomandnejad M., Nixon T. (2021). Novel Combinatorial MicroRNA-Binding Sites in AAV Vectors Synergistically Diminish Antigen Presentation and Transgene Immunity for Efficient and Stable Transduction. Front. Immunol..

[117] Li X., La Salvia S., Liang Y., Adamiak M., Kohlbrenner E., Jeong D., Chepurko E., Ceholski D., Lopez-Gordo E., Yoon S. (2023). Extracellular Vesicle–Encapsulated Adeno-Associated Viruses for Therapeutic Gene Delivery to the Heart. Circulation.

[118] György B., Maguire C.A. (2018). Extracellular vesicles: Nature’s nanoparticles for improving gene transfer with adeno-associated virus vectors. WIREs Nanomed. Nanobiotechnol..

[119] Guerra-Rebollo M., Stampa M., Lázaro M.Á., Cascante A., Fornaguera C., Borrós S. (2021). Electrostatic Coating of Viral Particles for Gene Delivery Applications in Muscular Dystrophies: Influence of Size on Stability and Antibody Protection. J. Neuromuscul. Dis..

[120] Cordova G., Negroni E., Cabello-Verrugio C., Mouly V., Trollet C. (2018). Combined Therapies for Duchenne Muscular Dystrophy to Optimize Treatment Efficacy. Front. Genet..

[121] Xin C., Chu X., Wei W., Kuang B., Wang Y., Tang Y., Chen J., You H., Li C., Wang B. (2021). Combined gene therapy via VEGF and mini-dystrophin synergistically improves pathologies in temporalis muscle of dystrophin/utrophin double knockout mice. Hum. Mol. Genet..

[122] Xu Y., Li Z. (2020). CRISPR-Cas systems: Overview, innovations and applications in human disease research and gene therapy. Comput. Struct. Biotechnol. J..

[123] Kampmann M. (2018). CRISPRi and CRISPRa Screens in Mammalian Cells for Precision Biology and Medicine. ACS Chem. Biol..

[124] Carroll M.S., Giacca M. (2023). CRISPR activation and interference as investigative tools in the cardiovascular system. Int. J. Biochem. Cell Biol..

[125] Jensen T.I., Mikkelsen N.S., Gao Z., Foßelteder J., Pabst G., Axelgaard E., Laustsen A., König S., Reinisch A., Bak R.O. (2021). Targeted regulation of transcription in primary cells using CRISPRa and CRISPRi. Genome Res..

[126] Omachi K., Miner J.H. (2022). Comparative analysis of dCas9-VP64 variants and multiplexed guide RNAs mediating CRISPR activation. PLoS ONE.

[127] Cheng A.W., Wang H., Yang H., Shi L., Katz Y., Theunissen T.W., Rangarajan S., Shivalila C.S., Dadon D.B., Jaenisch R. (2013). Multiplexed activation of endogenous genes by CRISPR-on, an RNA-guided transcriptional activator system. Cell Res..

[128] Gilbert L.A., Horlbeck M.A., Adamson B., Villalta J.E., Chen Y., Whitehead E.H., Guimaraes C., Panning B., Ploegh H.L., Bassik M.C. (2014). Genome-Scale CRISPR-Mediated Control of Gene Repression and Activation. Cell.

[129] Maeder M.L., Linder S.J., Cascio V.M., Fu Y., Ho Q.H., Joung J.K. (2013). CRISPR RNA–guided activation of endogenous human genes. Nat. Methods.

[130] Perez-Pinera P., Kocak D.D., Vockley C.M., Adler A.F., Kabadi A.M., Polstein L.R., Thakore P.I., Glass K.A., Ousterout D.G., Leong K.W. (2013). RNA-guided gene activation by CRISPR-Cas9–based transcription factors. Nat. Methods.

[131] Seipel K., Georgiev O., Schaffner W. (1992). Different activation domains stimulate transcription from remote (‘enhancer’) and proximal (‘promoter’) positions. Embo J..

[132] Konermann S., Brigham M.D., Trevino A.E., Joung J., Abudayyeh O.O., Barcena C., Hsu P.D., Habib N., Gootenberg J.S., Nishimasu H. (2015). Genome-scale transcriptional activation by an engineered CRISPR-Cas9 complex. Nature.

[133] Clark T., Waller M.A., Loo L., Moreno C.L., Denes C.E., Neely G.G. (2024). CRISPR activation screens: Navigating technologies and applications. Trends Biotechnol..

[134] Farzadfard F., Perli S.D., Lu T.K. (2013). Tunable and Multifunctional Eukaryotic Transcription Factors Based on CRISPR/Cas. ACS Synth. Biol..

[135] Gilbert L.A., Larson M.H., Morsut L., Liu Z., Brar G.A., Torres S.E., Stern-Ginossar N., Brandman O., Whitehead E.H., Doudna J.A. (2013). CRISPR-Mediated Modular RNA-Guided Regulation of Transcription in Eukaryotes. Cell.

[136] Kearns N.A., Genga R.M.J., Enuameh M.S., Garber M., Wolfe S.A., Maehr R. (2014). Cas9 effector-mediated regulation of transcription and differentiation in human pluripotent stem cells. Development.

[137] Perrin A., Rousseau J., Tremblay J.P. (2017). Increased Expression of Laminin Subunit Alpha 1 Chain by dCas9-VP160. Mol. Ther. Nucleic Acids.

[138] Ginley-Hidinger M., Carleton J.B., Rodriguez A.C., Berrett K.C., Gertz J. (2019). Sufficiency analysis of estrogen responsive enhancers using synthetic activators. Life Sci. Alliance.

[139] Balboa D., Weltner J., Eurola S., Trokovic R., Wartiovaara K., Otonkoski T. (2015). Conditionally Stabilized dCas9 Activator for Controlling Gene Expression in Human Cell Reprogramming and Differentiation. Stem Cell Rep..

[140] Tanenbaum M.E., Gilbert L.A., Qi L.S., Weissman J.S., Vale R.D. (2014). A Protein-Tagging System for Signal Amplification in Gene Expression and Fluorescence Imaging. Cell.

[141] Dominguez A.A., Chavez M.G., Urke A., Gao Y., Wang L., Qi L.S. (2022). CRISPR-Mediated Synergistic Epigenetic and Transcriptional Control. Cris. J..

[142] Zhou H., Liu J., Zhou C., Gao N., Rao Z., Li H., Hu X., Li C., Yao X., Shen X. (2018). In vivo simultaneous transcriptional activation of multiple genes in the brain using CRISPR–dCas9-activator transgenic mice. Nat. Neurosci..

[143] Swain T., Pflueger C., Freytag S., Poppe D., Pflueger J., Nguyen T.V., Li J.K., Lister R. (2024). A modular dCas9-based recruitment platform for combinatorial epigenome editing. Nucleic Acids Res..

[144] Kunii A., Hara Y., Takenaga M., Hattori N., Fukazawa T., Ushijima T., Yamamoto T., Sakuma T. (2018). Three-Component Repurposed Technology for Enhanced Expression: Highly Accumulable Transcriptional Activators via Branched Tag Arrays. Cris. J..

[145] Li Z., Zhang D., Xiong X., Yan B., Xie W., Sheen J., Li J.-F. (2017). A potent Cas9-derived gene activator for plant and mammalian cells. Nat. Plants.

[146] Young J.K., Gasior S.L., Jones S., Wang L., Navarro P., Vickroy B., Barrangou R. (2019). The repurposing of type I-E CRISPR-Cascade for gene activation in plants. Commun. Biol..

[147] Polstein L.R., Gersbach C.A. (2015). A light-inducible CRISPR-Cas9 system for control of endogenous gene activation. Nat. Chem. Biol..

[148] Becirovic E. (2022). Maybe you can turn me on: CRISPRa-based strategies for therapeutic applications. Cell. Mol. Life Sci..

[149] Böhm S., Riedmayr L.M., Nguyen O.N.P., Gießl A., Liebscher T., Butz E.S., Schön C., Michalakis S., Wahl-Schott C., Biel M. (2017). Peripherin-2 and Rom-1 have opposing effects on rod outer segment targeting of retinitis pigmentosa-linked peripherin-2 mutants. Sci. Rep..

[150] Liao H.K., Hatanaka F., Araoka T., Reddy P., Wu M.Z., Sui Y., Yamauchi T., Sakurai M., O’Keefe D.D., Núñez-Delicado E. (2017). In Vivo Target Gene Activation via CRISPR/Cas9-Mediated Trans-epigenetic Modulation. Cell.

[151] Wang G., Chow R.D., Bai Z., Zhu L., Errami Y., Dai X., Dong M.B., Ye L., Zhang X., Renauer P.A. (2019). Multiplexed activation of endogenous genes by CRISPRa elicits potent antitumor immunity. Nat. Immunol..

[152] Lek A., Wong B., Keeler A., Blackwood M., Ma K., Huang S., Sylvia K., Batista A.R., Artinian R., Kokoski D. (2023). Death after High-Dose rAAV9 Gene Therapy in a Patient with Duchenne’s Muscular Dystrophy. N. Engl. J. Med..

[153] U.S. Food and Drug Administration (2025). ZOLGENSMA (Onasemnogene Abeparvovec-xioi) Prescribing Information.

[154] Baranello G., Darras Basil T., Day John W., Deconinck N., Klein A., Masson R., Mercuri E., Rose K., El-Khairi M., Gerber M. (2021). Risdiplam in Type 1 Spinal Muscular Atrophy. N. Engl. J. Med..

[155] Mendell Jerry R., Al-Zaidy S., Shell R., Arnold W.D., Rodino-Klapac Louise R., Prior Thomas W., Lowes L., Alfano L., Berry K., Church K. (2017). Single-Dose Gene-Replacement Therapy for Spinal Muscular Atrophy. N. Engl. J. Med..

[156] Chen C.-D., Zeldich E., Li Y., Yuste A., Abraham C.R. (2018). Activation of the Anti-Aging and Cognition-Enhancing Gene Klotho by CRISPR-dCas9 Transcriptional Effector Complex. J. Mol. Neurosci..

[157] Wu R., Li P., Xiao P., Zhang S., Wang X., Liu J., Sun W., Chang Y., Ai X., Chen L. (2025). Activation of endogenous full-length utrophin by MyoAAV-UA as a therapeutic approach for Duchenne muscular dystrophy. Nat. Commun..

[158] Georgieva A.M., Guo X., Bartkuhn M., Günther S., Künne C., Smolka C., Atzberger A., Gärtner U., Mamchaoui K., Bober E. (2022). Inactivation of Sirt6 ameliorates muscular dystrophy in mdx mice by releasing suppression of utrophin expression. Nat. Commun..

[159] Qiao C., Yuan Z., Li J., He B., Zheng H., Mayer C., Li J., Xiao X. (2011). Liver-specific microRNA-122 target sequences incorporated in AAV vectors efficiently inhibits transgene expression in the liver. Gene Ther..

[160] Xin J., Liu S. (2025). Identifying hub genes and dysregulated pathways in Duchenne muscular dystrophy. Int. J. Neurosci..

[161] Wang J., Fan Q., Yu T., Zhang Y. (2021). Identifying the hub genes for Duchenne muscular dystrophy and Becker muscular dystrophy by weighted correlation network analysis. BMC Genom. Data.

[162] Suárez-Calvet X., Fernández-Simón E., Natera D., Jou C., Pinol-Jurado P., Villalobos E., Ortez C., Monceau A., Schiava M., Codina A. (2023). Decoding the transcriptome of Duchenne muscular dystrophy to the single nuclei level reveals clinical-genetic correlations. Cell Death Dis..

[163] Sothers H., Hu X., Crossman D.K., Si Y., Alexander M.S., McDonald M.-L.N., King P.H., Lopez M.A. (2024). Late-Stage Skeletal Muscle Transcriptome in Duchenne muscular dystrophy shows a BMP4-Induced Molecular Signature. bioRxiv.

[164] Kotterman M.A., Schaffer D.V. (2014). Engineering adeno-associated viruses for clinical gene therapy. Nat. Rev. Genet..

[165] Labbé R.P., Vessillier S., Rafiq Q.A. (2021). Lentiviral Vectors for T Cell Engineering: Clinical Applications, Bioprocessing and Future Perspectives. Viruses.

[166] Lan T., Que H., Luo M., Zhao X., Wei X. (2022). Genome editing via non-viral delivery platforms: Current progress in personalized cancer therapy. Mol. Cancer.

[167] Lambricht L., Lopes A., Kos S., Sersa G., Préat V., Vandermeulen G. (2016). Clinical potential of electroporation for gene therapy and DNA vaccine delivery. Expert Opin. Drug Deliv..

[168] Li F., Wing K., Wang J.-H., Luu C.D., Bender J.A., Chen J., Wang Q., Lu Q., Nguyen Tran M.T., Young K.M. (2020). Comparison of CRISPR/Cas Endonucleases for in vivo Retinal Gene Editing. Front. Cell. Neurosci..

[169] Nelson C.E., Gersbach C.A. (2016). Engineering Delivery Vehicles for Genome Editing. Annu. Rev. Chem. Biomol. Eng..

[170] Yang Z.-X., Fu Y.-W., Zhao J.-J., Zhang F., Li S.-A., Zhao M., Wen W., Zhang L., Cheng T., Zhang J.-P. (2023). Superior Fidelity and Distinct Editing Outcomes of SaCas9 Compared with SpCas9 in Genome Editing. Genom. Proteom. Bioinform..

[171] Yang H., Patel D.J. (2019). CasX: A new and small CRISPR gene-editing protein. Cell Res..

[172] Nakagawa R., Ishiguro S., Okazaki S., Mori H., Tanaka M., Aburatani H., Yachie N., Nishimasu H., Nureki O. (2022). Engineered Campylobacter jejuni Cas9 variant with enhanced activity and broader targeting range. Commun. Biol..

[173] Gleditzsch D., Pausch P., Müller-Esparza H., Özcan A., Guo X., Bange G., Randau L. (2019). PAM identification by CRISPR-Cas effector complexes: Diversified mechanisms and structures. RNA Biol..

[174] Ibreljic N., Draper B.E., Lawton C.W. (2024). Recombinant AAV genome size effect on viral vector production, purification, and thermostability. Mol. Ther.-Methods Clin. Dev..

[175] Trapani I., Colella P., Sommella A., Iodice C., Cesi G., de Simone S., Marrocco E., Rossi S., Giunti M., Palfi A. (2014). Effective delivery of large genes to the retina by dual AAV vectors. EMBO Mol. Med..

[176] Truong D.-J.J., Kühner K., Kühn R., Werfel S., Engelhardt S., Wurst W., Ortiz O. (2015). Development of an intein-mediated split–Cas9 system for gene therapy. Nucleic Acids Res..

[177] He X., Urip B.A., Zhang Z., Ngan C.C., Feng B. (2021). Evolving AAV-delivered therapeutics towards ultimate cures. J. Mol. Med..

[178] Servais L., Horton R., Saade D., Bonnemann C., Muntoni F., Adjali D.O., Beggs D.A., Bharucha D.D., Bönnemann D.C., Braun D.S. (2023). 261st ENMC International Workshop: Management of safety issues arising following AAV gene therapy. 17th-19th June 2022, Hoofddorp, The Netherlands. Neuromuscul. Disord..

[179] Manini A., Abati E., Nuredini A., Corti S., Comi G.P. (2021). Adeno-Associated Virus (AAV)-Mediated Gene Therapy for Duchenne Muscular Dystrophy: The Issue of Transgene Persistence. Front. Neurol..

[180] Tsuchida C.A., Zhang S., Doost M.S., Zhao Y., Wang J., O’Brien E., Fang H., Li C.-P., Li D., Hai Z.-Y. (2022). Chimeric CRISPR-CasX enzymes and guide RNAs for improved genome editing activity. Mol. Cell.

[181] Tabebordbar M., Lagerborg K.A., Stanton A., King E.M., Ye S., Tellez L., Krunnfusz A., Tavakoli S., Widrick J.J., Messemer K.A. (2021). Directed evolution of a family of AAV capsid variants enabling potent muscle-directed gene delivery across species. Cell.

[182] Dhungel B.P., Winburn I., Pereira C.D.F., Huang K., Chhabra A., Rasko J.E.J. (2024). Understanding AAV vector immunogenicity: From particle to patient. Theranostics.

[183] Ertl H.C.J. (2022). Immunogenicity and toxicity of AAV gene therapy. Front. Immunol..

[184] Bing S.J., Seirup M., Hoang T.T., Najera S.S., Britten C., Warrington S.L., Chu S.L., Mazor R. (2024). Rational immunosilencing of a promiscuous T-cell epitope in the capsid of an adeno-associated virus. Nat. Biomed. Eng..

[185] Bentler M., Hardet R., Ertelt M., Rudolf D., Kaniowska D., Schneider A., Vondran F.W.R., Schoeder C.T., Delphin M., Lucifora J. (2023). Modifying immune responses to adeno-associated virus vectors by capsid engineering. Mol. Ther. Methods Clin. Dev..

[186] Barnes C., Scheideler O., Schaffer D. (2019). Engineering the AAV capsid to evade immune responses. Curr. Opin. Biotechnol..

[187] Majeau N., Fortin-Archambault A., Gérard C., Rousseau J., Yaméogo P., Tremblay J.P. (2022). Serum extracellular vesicles for delivery of CRISPR-CAS9 ribonucleoproteins to modify the dystrophin gene. Mol. Ther..

[188] Kenjo E., Hozumi H., Makita Y., Iwabuchi K.A., Fujimoto N., Matsumoto S., Kimura M., Amano Y., Ifuku M., Naoe Y. (2021). Low immunogenicity of LNP allows repeated administrations of CRISPR-Cas9 mRNA into skeletal muscle in mice. Nat. Commun..

[189] Xu J., Xu J., Sun C., He X., Shu Y., Huangfu Q., Meng L., Liang Z., Wei J., Cai M. (2025). Effective delivery of CRISPR/dCas9-SAM for multiplex gene activation based on mesoporous silica nanoparticles for bladder cancer therapy. Acta Biomater..

[190] Gee P., Lung M.S.Y., Okuzaki Y., Sasakawa N., Iguchi T., Makita Y., Hozumi H., Miura Y., Yang L.F., Iwasaki M. (2020). Extracellular nanovesicles for packaging of CRISPR-Cas9 protein and sgRNA to induce therapeutic exon skipping. Nat. Commun..

[191] Wei T., Cheng Q., Min Y.-L., Olson E.N., Siegwart D.J. (2020). Systemic nanoparticle delivery of CRISPR-Cas9 ribonucleoproteins for effective tissue specific genome editing. Nat. Commun..

[192] Wang J., Ding Y., Chong K., Cui M., Cao Z., Tang C., Tian Z., Hu Y., Zhao Y., Jiang S. (2024). Recent Advances in Lipid Nanoparticles and Their Safety Concerns for mRNA Delivery. Vaccines.

[193] Jung H.N., Lee S.Y., Lee S., Youn H., Im H.J. (2022). Lipid nanoparticles for delivery of RNA therapeutics: Current status and the role of in vivo imaging. Theranostics.

[194] Kularatne R.N., Crist R.M., Stern S.T. (2022). The Future of Tissue-Targeted Lipid Nanoparticle-Mediated Nucleic Acid Delivery. Pharmaceuticals.

[195] Shoji E., Sakurai H., Nishino T., Nakahata T., Heike T., Awaya T., Fujii N., Manabe Y., Matsuo M., Sehara-Fujisawa A. (2015). Early pathogenesis of Duchenne muscular dystrophy modelled in patient-derived human induced pluripotent stem cells. Sci. Rep..

[196] Caputo L., Granados A., Lenzi J., Rosa A., Ait-Si-Ali S., Puri P.L., Albini S. (2020). Acute conversion of patient-derived Duchenne muscular dystrophy iPSC into myotubes reveals constitutive and inducible over-activation of TGFβ-dependent pro-fibrotic signaling. Skelet. Muscle.

[197] Palmieri L., Pili L., Jaber A., Hong A.V., Moula M., El-Khoury R., Brochiet G., Bigot A., Israeli D., Richard I. (2024). Disease exacerbation in MYOrganoids derived from Duchenne Muscular Dystrophy iPSC reveals limitations of microdystrophin therapeutic efficacy. bioRxiv.

[198] Partridge T.A. (2013). The mdx mouse model as a surrogate for Duchenne muscular dystrophy. FEBS J..

[199] Egorova T.V., Zotova E.D., Reshetov D.A., Polikarpova A.V., Vassilieva S.G., Vlodavets D.V., Gavrilov A.A., Ulianov S.V., Buchman V.L., Deykin A.V. (2019). CRISPR/Cas9-generated mouse model of Duchenne muscular dystrophy recapitulating a newly identified large 430 kb deletion in the human DMD gene. Dis. Models Mech..

[200] Young C.S., Mokhonova E., Quinonez M., Pyle A.D., Spencer M.J. (2017). Creation of a Novel Humanized Dystrophic Mouse Model of Duchenne Muscular Dystrophy and Application of a CRISPR/Cas9 Gene Editing Therapy. J. Neuromuscul. Dis..

[201] van Putten M., Hulsker M., Young C., Nadarajah V.D., Heemskerk H., van der Weerd L., t Hoen P.A., van Ommen G.J., Aartsma-Rus A.M. (2013). Low dystrophin levels increase survival and improve muscle pathology and function in dystrophin/utrophin double-knockout mice. FASEB J..

[202] Taglietti V., Kefi K., Bronisz-Budzyńska I., Mirciloglu B., Rodrigues M., Cardone N., Coulpier F., Periou B., Gentil C., Goddard M. (2022). Duchenne muscular dystrophy trajectory in R-DMDdel52 preclinical rat model identifies COMP as biomarker of fibrosis. Acta Neuropathol. Commun..

[203] Larcher T., Lafoux A., Tesson L., Remy S., Thepenier V., François V., Le Guiner C., Goubin H., Dutilleul M., Guigand L. (2014). Characterization of dystrophin deficient rats: A new model for Duchenne muscular dystrophy. PLoS ONE.

[204] Nakamura K., Fujii W., Tsuboi M., Tanihata J., Teramoto N., Takeuchi S., Naito K., Yamanouchi K., Nishihara M. (2014). Generation of muscular dystrophy model rats with a CRISPR/Cas system. Sci. Rep..

[205] Szabó P.L., Ebner J., Koenig X., Hamza O., Watzinger S., Trojanek S., Abraham D., Todt H., Kubista H., Schicker K. (2021). Cardiovascular phenotype of the Dmd(mdx) rat—a suitable animal model for Duchenne muscular dystrophy. Dis. Model. Mech..

[206] Otake M., Imamura M., Enya S., Kangawa A., Shibata M., Ozaki K., Kimura K., Ono E., Aoki Y. (2024). Severe cardiac and skeletal manifestations in DMD-edited microminipigs: An advanced surrogate for Duchenne muscular dystrophy. Commun. Biol..

[207] Stirm M., Klymiuk N., Nagashima H., Kupatt C., Wolf E. (2024). Pig models for translational Duchenne muscular dystrophy research. Trends Mol. Med..

[208] Kornegay J.N., Bogan J.R., Bogan D.J., Childers M.K., Li J., Nghiem P., Detwiler D.A., Larsen C.A., Grange R.W., Bhavaraju-Sanka R.K. (2012). Canine models of Duchenne muscular dystrophy and their use in therapeutic strategies. Mamm. Genome.

[209] Barthélémy I., Calmels N., Weiss R.B., Tiret L., Vulin A., Wein N., Peccate C., Drougard C., Beroud C., Deburgrave N. (2020). X-linked muscular dystrophy in a Labrador Retriever strain: Phenotypic and molecular characterisation. Skelet. Muscle.

[210] Charlesworth C.T., Deshpande P.S., Dever D.P., Camarena J., Lemgart V.T., Cromer M.K., Vakulskas C.A., Collingwood M.A., Zhang L., Bode N.M. (2019). Identification of preexisting adaptive immunity to Cas9 proteins in humans. Nat. Med..

[211] Crudele J.M., Chamberlain J.S. (2018). Cas9 immunity creates challenges for CRISPR gene editing therapies. Nat. Commun..

[212] Roy S., Ghosh D. (2021). Chapter Eight—Immune responses to CRISPR-Cas protein. Progress in Molecular Biology and Translational Science.

[213] Heidersbach A.J., Dorighi K.M., Gomez J.A., Jacobi A.M., Haley B. (2023). A versatile, high-efficiency platform for CRISPR-based gene activation. Nat. Commun..

